# Survival of the Littlest: Navigating Sepsis Diagnosis beyond Inflammation in Preterm Neonates

**DOI:** 10.1021/acs.jproteome.4c01072

**Published:** 2025-04-30

**Authors:** Manchu Umarani Thangavelu, Alida Kindt, Shawen Hassan, Jelte J. B. Geerlings, Charlotte Nijgh-van Kooij, Irwin K. M. Reiss, Bert Wouters, H. Rob Taal, Thomas Hankemeier

**Affiliations:** † Metabolomics and Analytics Center, 4496 Leiden University, 2333 CC Leiden, The Netherlands; ‡ Department of Neonatal and Pediatric Intensive Care, Division of Neonatology, 6993 Erasmus MC, 3000 CB Rotterdam, The Netherlands

**Keywords:** bacterial infection, late-onset sepsis, metabolomics, preterm, systemic inflammation

## Abstract

Sepsis diagnosis in preterm neonates is challenging due to symptom overlap with non-infectious inflammatory conditions, and slow, unreliable diagnostic practices. This case-control study aims to elucidate sepsis pathophysiology, and identify metabolic biomarkers for timely, accurate diagnosis, to prevent rapid health deterioration and unnecessary antibiotic use. Liquid chromatography–mass spectrometry was performed on 227 plasma samples, obtained from 94 preterm neonates, to measure 317 metabolites encompassing amines and signaling lipids. Linear mixed-effect modeling, LASSO and logistic regression models were calculated to assess metabolic alterations across control, systemic inflammation-no sepsis (SINS), and sepsis groups. Stratification by sex and pathogen type allowed identification of sex-specific responses and pathogen-driven variations in sepsis. Key findings include (i) shared metabolic changes in SINS and sepsis, (ii) progressive alterations from control to SINS to sepsis, and (iii) sepsis-specific markers. Males exhibited a pro-inflammatory phenotype while females showed an anti-inflammatory phenotype in response to sepsis. Gram-positive and gram-negative bacterial sepsis revealed distinct metabolic profiles. A diagnostic model comprising 5 metabolic features and IL-6 distinguished SINS from sepsis at clinical suspicion (AUC 0.79, sensitivity 0.85, specificity 0.82). These insights highlight the potential of metabolomics to revolutionize neonatal sepsis management with precision diagnostics and personalized treatment strategies.

## Graphical abstract

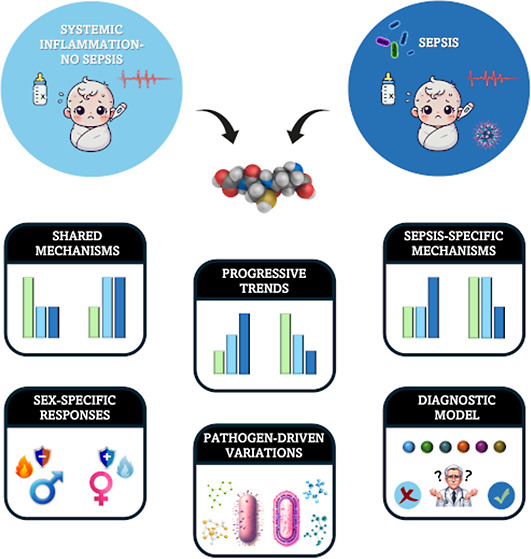

## 1 Introduction

Preterm birth, defined as delivery before 37 weeks of gestation, accounts for approximately 1 in 10 live births globally.^1^ While advancements in antenatal care have not significantly reduced the global preterm birth rate, postnatal care interventions have increased the survival probability of even the extremely preterm neonates (<28 weeks gestational age).^1,2^ However, complications associated with prematurity remain the leading cause of adverse health outcomes and under-five mortality.^3,4^ A major contributor to neonatal mortality and long-term morbidity is neonatal sepsis, a systemic infection of microbial origin that occurs within the first month of life.^5^ Neonatal sepsis affects an estimated 1.3 million neonates annually worldwide resulting in ∼203,000 deaths, with higher incidence and mortality rates in males compared to females.^5,6^ Time of onset classifies neonatal sepsis as early onset sepsis, occurring within 72 h of life typically from bacterial infections vertically transmitted during birth, or late-onset sepsis (LOS) which develops after 72 h from postnatal environmental exposures.^5^ Early onset sepsis incidence has declined over the years due to advanced screening and intrapartum antibiotic prophylaxis, but LOS rates have remained unchanged, posing a persistent burden to neonatal healthcare.^5^

The third international consensus defines sepsis as a life-threatening organ dysfunction caused by a dysregulated host response to an infection, providing a valuable diagnostic framework for adults.^7^ However, neonatal sepsis presents with a multitude of inconclusive symptoms, such as abnormal vital signs and feeding intolerance, and gestational- and postnatal-age-dependent variability in clinical presentations, which necessitates a comprehensive approach incorporating various clinical criteria, laboratory markers, and microbiological data (Figure 1a).^5,8^ Although blood cultures are the golden standard for sepsis confirmation, their turnover time of ∼48 h precludes timely interventions, and non-culturable or insufficient live pathogens in samples return false culture-negative results.^5^ Inflammatory biomarkers such as c-reactive protein (CRP), interleukin-6 (IL-6), and procalcitonin (PCT), offer a robust negative predictive value to rule out infections but lack sensitivity and specificity to accurately distinguish sepsis from other non-infectious inflammatory conditions.^5^ Compounding the challenge, sepsis caused by gram-negative bacteria often presents with more severe symptoms and higher mortality than gram-positive bacteria, compelling the empirical use of broad-spectrum antibiotics to all neonates upon sepsis suspicion (Figure 1a).^5,9^ However, this practice risks unnecessary antibiotic use, contributing to multidrug-resistant pathogens and gut microbiome disruption which may result in long-term conditions like obesity and asthma.^10^ Therefore, early identification of causative pathogens is crucial for tailoring antibiotic therapy and mitigating these risks.

**Figure 1:**
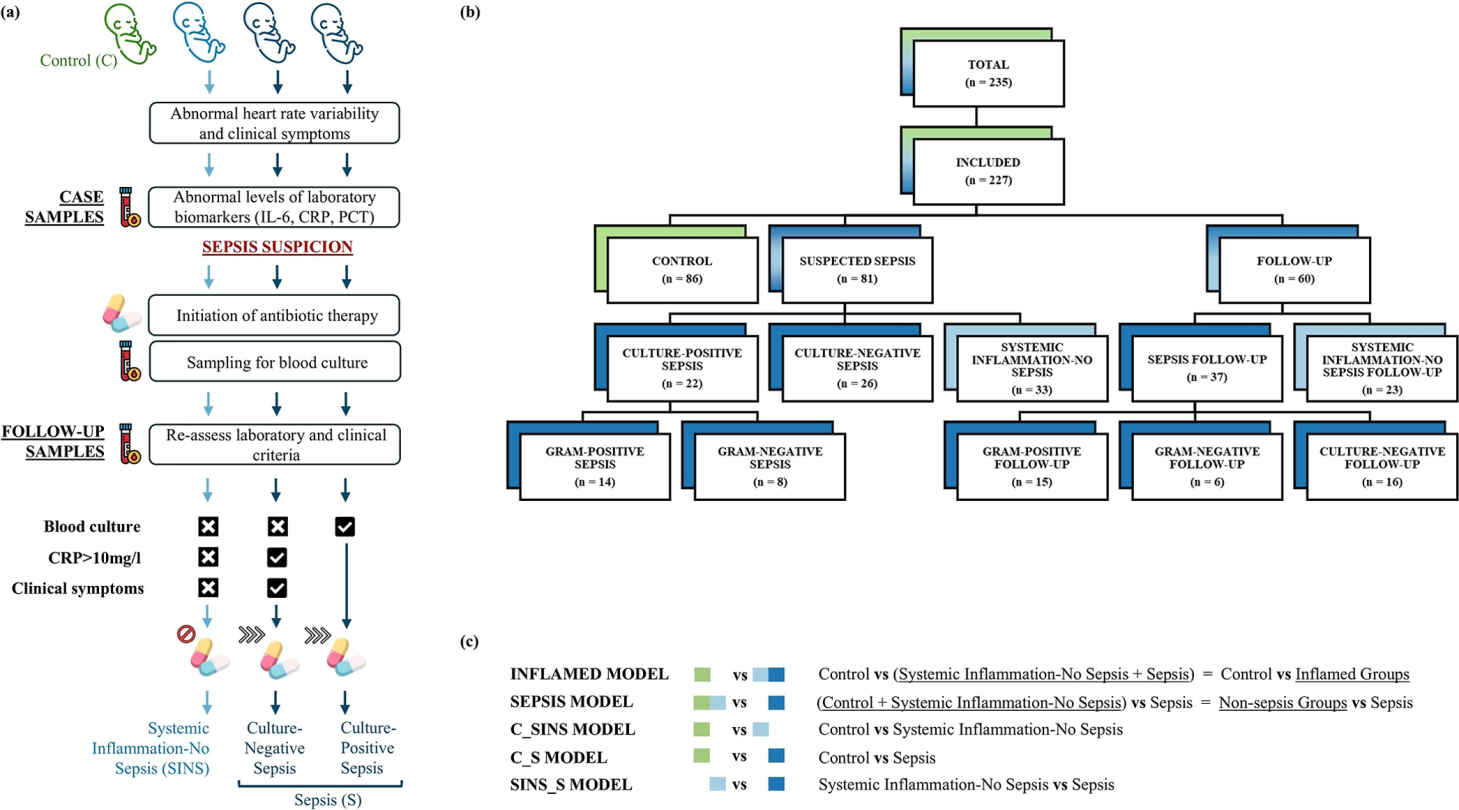
Overview of sample classification criteria, cohort composition, and metabolic modeling framework. (a) The flowchart illustrates the clinical trajectories and criteria for classifying neonates into control, systemic inflammation-no sepsis, culture-negative sepsis and culture-positive sepsis groups, based on clinical symptoms, laboratory findings, and microbiological results. (b) The flowchart depicts the cohort composition, with samples initially classified into control, suspected sepsis, and follow-up samples. For statistical analyses, all samples were grouped into three distinct categories: control (green; *n* = 86), systemic inflammation-no sepsis (light blue; *n* = 56), and sepsis (blue; *n* = 85), wherein the follow-up samples were reclassified into either sepsis or systemic inflammation-no sepsis based on their initial diagnosis. (c) The primary models compared Control vs Inflamed groups and Non-Sepsis groups vs Sepsis to identify systemic inflammation- and sepsis-specific traits. Additional pairwise comparisons were performed between groups: Control vs Systemic Inflammation-No Sepsis (C_SINS), Control vs Sepsis (C_S), and Systemic Inflammation-No Sepsis vs Sepsis (SINS_S). IL-6 = Interleukin-6; CRP = C-Reactive Protein; PCT = Procalcitonin. Icons used in the figure are from Flaticon (www.flaticon.com).

Metabolomics offers a promising avenue to elucidate sepsis pathophysiology and discover novel biomarkers to complement traditional diagnostic methods. Metabolism plays a vital role in immune cell differentiation and function, influencing both the effector and resolution phases of inflammation. Key pathways, including glycolysis, oxidative phosphorylation, fatty acid (FA) oxidation, and amino acid metabolism, have been implicated in immune regulation.^11^ The reconfiguration of immunometabolism during inflammatory disorders thus provides insights into disease mechanisms and outcomes. Metabolomic studies investigating LOS in preterm neonates are scarce but have unveiled significant disruptions of biological pathways and imbalances in biochemical homeostasis.^12–16^ However, these studies often compare sepsis cases with controls showing no signs of sepsis or lack clear specification of the inclusion/exclusion criteria for the groups under comparison. This methodological limitation overlooks the critical aspect of identifying sepsis among other inflammatory conditions exhibiting sepsis-like symptoms, which is essential for accurate sepsis diagnosis.

Our research aims to bridge this gap by comparing the metabolic profiles of preterm neonates across three groups: neonates without inflammation, neonates with suspected LOS later diagnosed as non-septic, and neonates diagnosed and treated for LOS including both culture-positive and culture-negative cases. In a targeted LC–MS approach, 296 metabolic features covering oxylipins, endocannabinoids, lysophospholipids, free FAs, bile acids, and amines were analyzed in plasma samples to elucidate the similarities and differences in metabolic signatures among these groups. The analyses aim to (i) enhance our understanding of the underlying pathophysiology of sepsis, (ii) identify potential diagnostic biomarkers, and (iii) explore potential sex-specific and pathogen-specific differences in metabolic profiles to provide valuable insights into the heterogeneity of sepsis presentations and responses. These investigations will aid the development of improved diagnostic and tailored therapeutic strategies for LOS in preterm neonates.

## 2 Methods

### 2.1 Cohort

The study included 94 preterm neonates (gestational ages: 24–32 weeks) admitted to the Erasmus MC Sophia Children’s Hospital in The Netherlands, between August 2021 and November 2022. A total of 235 samples were categorized into control, suspected sepsis, and follow-up groups. Suspected sepsis samples were obtained upon clinical suspicion of sepsis based on symptoms and heart rate characteristics index,^17^ with follow-up samples collected 6–48 h later. Control samples, matched for gestational age (±1 week), sex, and postnatal age (±4 days), showed no signs of sepsis. The suspected sepsis samples were further classified into “culture-positive sepsis”, “culture-negative sepsis”, or “no sepsis” based on clinical and laboratory findings. Hereafter, the “no sepsis” group is referred to as “Systemic Inflammation-No Sepsis” (SINS) to underscore the systemic inflammatory response observed in the absence of pathogens that initially prompted sepsis suspicion. An overview of the clinical trajectories and classification criteria for these groups is provided in Figure 1a and Supporting Information S1.

Analyses were conducted on two data sets: a comprehensive data set for elucidating sepsis pathophysiology and a single-time point (STP) data set for identifying potential early diagnostic biomarkers. The comprehensive data set incorporated all available samples, consolidating follow-up samples with their corresponding groups (sepsis or SINS) to enhance statistical power (Figure 1b). The STP data set was curated to include only one sample per patient by excluding follow-up samples, prioritizing first episodes of sepsis cases followed by SINS cases, and including matched controls exclusively from neonates without any occurrences of SINS or sepsis. Figure S1 illustrates the selection of STP data set samples from the comprehensive data set, plotted by the postnatal age at time of sampling.

### 2.2 Sample Collection

Blood samples (500 μL) were collected via an arterial line or heel prick, processed immediately to obtain plasma, and partially utilized for serological analyses, including CRP, IL-6, and PCT measurements. The remaining plasma was stored in a biobank at −80 °C within 24 h, then later retrieved and transported on dry ice to the analytical chemistry laboratory, where it was stored at −80 °C until aliquoting for metabolomic analysis (Supporting Information S1).

### 2.3 Metabolomic Data Acquisition

Amino acids, biogenic amines, and signaling lipids were quantified in plasma using ultra-performance liquid- chromatography tandem mass-spectrometry. Plasma samples were prepared through the addition of internal standards, followed by protein precipitation and derivatization for amines or lipid extraction for signaling lipids. Chromatographic separations were carried out on Agilent and Shimadzu systems equipped with C18 columns, and analytes were detected using multiple reaction monitoring on Sciex QTRAP mass spectrometers. Data acquisition and peak integration were performed using Sciex MultiQuant and Sciex OS software. A detailed description of the experimental protocols is provided in the Supporting Information S2.

### 2.4 Data Preprocessing

Quality control was performed by retaining only metabolites with <30% relative standard deviation in pooled study samples, <40% background signal in method blanks, and <20% missingness. Biologically pertinent metabolite ratios, sums, and means were computed to provide insights into enzyme activities and shifts in metabolic pathways. All data were log_2_ transformed, missing values imputed using the Quantile Regression Imputation of Left-Censored data method,^18^ and auto-scaled for multivariable analysis. A list of all included metabolic features and their abbreviations is provided in Table S1. Further details are provided in Supporting Information S3.

### 2.5 Statistics

All statistical analyses were performed in RStudio (v4.3.1). Linear mixed models (LMMs), ANOVA, and fold change (FC) analyses were applied to the comprehensive data set to identify metabolic associations with controls, SINS, and sepsis. Two primary LMMs were implemented: (i) the “Inflamed” model, comparing the inflamed groups (SINS + sepsis) to controls, and (ii) the “Sepsis” model, comparing the non-sepsis groups (control + SINS) to sepsis. Additional pairwise comparisons (control vs SINS (C_SINS model), control vs sepsis (C_S model), SINS vs sepsis (SINS_S model)) were used to aid interpretation. LMM results were visualized using volcano plots and compared using forest plots and directed *p*-value plots, where the directed *p*-values are the negative logarithm (base 10) of the *p*-value multiplied with the sign of the estimate. Further analyses investigated the Sepsis model to data sets stratified by sex, pathogen, and blood culture outcome. All models were corrected for confounders identified by Fisher’s exact test for categorical variables, Welch’s *t*-test for continuous variables, and visual inspection by principal component analysis (PCA).

The STP data set was investigated using logistic regression, bootstrap-aggregated LASSO logistic regression, spearman and differential correlation analyses. Briefly, logistic regression univariate models were computed for pairwise comparisons (C_SINS, C_S, and SINS_S) to investigate the diagnostic potential of metabolites. Bootstrap-aggregated LASSO logistic regression was used to identify multivariable metabolomic diagnostic models to distinguish between SINS and sepsis at the moment of suspicion. Predictive performance was estimated by averaging metrics across 100 bootstrap iterations. A reduced model based on consistently selected features across bootstraps was evaluated using Leave-One-Out Cross-Validation to derive the area under the receiving operating characteristics curve and compared against inflammatory marker and integrated models using McNemar’s test. Furthermore, spearman and differential correlation analyses were performed to explore the relationship between metabolites and inflammatory markers under SINS and sepsis. A significance threshold of 0.05 was applied to *p*-values across all statistical analyses. To account for multiple testing in univariate analyses, *p*-values were adjusted using Benjamini–Hochberg method applying a significance threshold of *q* < 0.1. These corrections accounted for the total number of metabolic features in univariate tests (*n* = 296). A detailed description of the analyses is provided in Supporting Information S4.

### 2.6 Ethics

The study was approved by the local ethics committee of the Erasmus Medical Centre (MEC-2020-0547). In accordance with standard care protocols, informed parental consent for the utilization of data and residual biomaterial for future medical research was obtained upon patients’ admission to the neonatal intensive care unit.

## 3 Results

### 3.1 Cohort Characteristics

After quality control, 8 of 235 samples were excluded from analysis; 1 due to diagnostic ambiguity from misclassification, 1 due to insufficient sample volume, and 6 identified as outliers - 4 via the Rosner test based on internal standards and 2 through visual inspection during peak integration and PCA. Of 317 metabolites measured using two assays, 254 met the predefined acceptance criteria, with 208 presenting varying percentages of missingness. Forty metabolites had missing data exceeding 20%, with missingness patterns detailed in Table S2. The study included 10 confounders: sample source (artery/capillary), mechanical ventilation, intraventricular hemorrhage, and antibiotic administration within 24 h prior to sampling were found to be associated with sepsis outcome, while gestational age, birth weight, postnatal age, sex, oxygen saturation, and proportion of calories provided by total parenteral nutrition were found to influence the metabolite levels.^19–21^ A summary of key demographic and clinical characteristics across both comprehensive and STP data sets, is provided in Table 1a,b.

**Table 1:** Demographic and Clinical Characteristics of the Study Cohort^a^

		Comprehensive Data Set (*n* = 227)							
					sepsis (*n* = 85)				
							culture-positive sepsis (*n* = 43)		
characteristic		all (*n* = 227)	control (*n* = 86)	systemic inflammation-no sepsis (*n* = 56)	all (*n* = 85)	culture-negative sepsis (*n* = 42)	all (*n* = 43)	gram-positive sepsis (*n* = 29)	gram-negative sepsis (*n* = 14)
gestational age at birth in weeks [mean ± sd]		26.67 ± 2.07	26.8 ± 2.01	26.41 ± 1.95	26.71 ± 2.23	26.48 ± 2.29	26.93 ± 2.17	27.17 ± 2.34	26.42 ± 1.74
birthweight in grams [mean ± sd]		891.9 ± 279.45	926.66 ± 259.7	813.04 ± 249.22	908.68 ± 309.06	863.05 ± 277.41	953.26 ± 334.35	978.97 ± 385.16	900 ± 192.69
current weight at sampling in grams [mean ± sd]		1137.89 ± 417.86	1169.76 ± 434.5	1053.54 ± 329.72	1161.24 ± 448.58	1199.05 ± 396.08	1124.3 ± 496.45	1143.45 ± 525.23	1084.64 ± 446.76
postnatal age at sampling in days [mean ± sd]		21.19 ± 18.45	21.03 ± 19.06	21.05 ± 18.78	21.45 ± 17.81	26.76 ± 18.86	16.26 ± 15.21	14.97 ± 13.2	18.93 ± 18.99
proportion of total parenteral nutrition [mean ± sd]		0.38 ± 0.38	0.36 ± 0.36	0.38 ± 0.42	0.4 ± 0.38	0.36 ± 0.42	0.45 ± 0.34	0.48 ± 0.34	0.39 ± 0.35
percentage of oxygen level [mean ± sd]		30.13 ± 14.33	26.27 ± 8.28	32.75 ± 19.13	32.31 ± 14.8	31.5 ± 11.47	33.09 ± 17.56	34.55 ± 18.18	30.07 ± 16.42
gender [*n* (%)]	male	136 (59.91)	52 (60.47)	29 (51.79)	55 (64.71)	22 (52.38)	33 (76.74)	22 (75.86)	11 (78.57)
	female	91 (40.09)	34 (39.53)	27 (48.21)	30 (35.29)	20 (47.62)	10 (23.26)	7 (24.14)	3 (21.43)
sample origin [*n* (%)]	artery	51 (22.47)	9 (10.47)	15 (26.79)	27 (31.76)	11 (26.19)	16 (37.21)	13 (44.83)	3 (21.43)
	capillary	176 (77.53)	77 (89.53)	41 (73.21)	58 (68.24)	31 (73.81)	27 (62.79)	16 (55.17)	11 (78.57)
mechanical ventilation [*n* (%)]	no	115 (50.66)	58 (67.44)	27 (48.21)	30 (35.29)	10 (23.81)	20 (46.51)	12 (41.38)	8 (57.14)
	yes	112 (49.34)	28 (32.56)	29 (51.79)	55 (64.71)	32 (76.19)	23 (53.49)	17 (58.62)	6 (42.86)
intraventricular hemorrhage [*n* (%)]	no	166 (73.13)	71 (82.56)	46 (82.14)	49 (57.65)	28 (66.67)	21 (48.84)	13 (44.83)	8 (57.14)
	yes	61 (26.87)	15 (17.44)	10 (17.86)	36 (42.35)	14 (33.33)	22 (51.16)	16 (55.17)	6 (42.86)

	Single Time Point Data Set (*n* = 92)								
					sepsis (*n* = 40)				
							culture-positive sepsis (*n* = 19)		
characteristic		all (*n* = 92)	control (*n* = 35)	systemic inflammation-no sepsis (*n* = 17)	all (*n* = 40)	culture-negative sepsis (*n* = 21)	all (*n* = 19)	gram-positive sepsis (*n* = 12)	gram-negative sepsis (*n* = 7)
gestational age at birth in weeks [mean ± sd]		27.09 ± 2.1	27.67 ± 2.09	27.23 ± 1.7	26.51 ± 2.17	26.42 ± 2.09	26.61 ± 2.3	27.02 ± 2.56	25.9 ± 1.71
birthweight in grams [mean ± sd]		958.85 ± 289.58	1055.74 ± 269.66	908.82 ± 235.95	895.32 ± 309.49	844.9 ± 260.82	951.05 ± 354.54	1022.92 ± 414.71	827.86 ± 183.69
current weight at sampling in grams [mean ± sd]		1118.16 ± 432.58	1228.29 ± 488.98	968.88 ± 272.66	1085.25 ± 419.5	1080 ± 301.51	1091.05 ± 529.35	1085 ± 502.77	1101.43 ± 614.01
postnatal age at sampling in days [mean ± sd]		16.49 ± 16.92	16.6 ± 19.85	11.35 ± 9.33	18.58 ± 16.53	22.38 ± 14.85	14.37 ± 17.64	10.58 ± 8.9	20.86 ± 26.66
proportion of total parenteral nutrition [mean ± sd]		0.39 ± 0.35	0.44 ± 0.35	0.43 ± 0.39	0.32 ± 0.34	0.26 ± 0.37	0.39 ± 0.32	0.44 ± 0.33	0.3 ± 0.3
percentage of oxygen level [mean ± sd]		28.27 ± 12.33	24.23 ± 7.99	28 ± 15.96	31.92 ± 12.89	30.86 ± 9.89	33.11 ± 15.76	32.17 ± 12.45	34.71 ± 21.36
gender [*n* (%)]	male	52 (56.52)	18 (51.43)	8 (47.06)	26 (65)	12 (57.14)	14 (73.68)	9 (75)	5 (71.43)
	female	40 (43.48)	17 (48.57)	9 (52.94)	14 (35)	9 (42.86)	5 (26.32)	3 (25)	2 (28.57)
sample origin [*n* (%)]	artery	19 (20.65)	3 (8.57)	4 (23.53)	12 (30)	5 (23.81)	7 (36.84)	6 (50)	1 (14.29)
	capillary	73 (79.35)	32 (91.43)	13 (76.47)	28 (70)	16 (76.19)	12 (63.16)	6 (50)	6 (85.71)
mechanical ventilation [*n* (%)]	no	54 (58.7)	26 (74.29)	11 (64.71)	17 (42.5)	8 (38.1)	9 (47.37)	5 (41.67)	4 (57.14)
	yes	38 (41.3)	9 (25.71)	6 (35.29)	23 (57.5)	13 (61.9)	10 (52.63)	7 (58.33)	3 (42.86)
intraventricular hemorrhage [*n* (%)]	no	68 (73.91)	31 (88.57)	14 (82.35)	23 (57.5)	13 (61.9)	10 (52.63)	5 (41.67)	5 (71.43)
	yes	24 (26.09)	4 (11.43)	3 (17.65)	17 (42.5)	8 (38.1)	9 (47.37)	7 (58.33)	2 (28.57)

a The table presents the demographic and clinical characteristics of the (a) comprehensive dataset and (b) single time point dataset, including gestational age, birth weight, current weight, postnatal age, proportion of total parenteral nutrition, oxygen saturation percentage, sex distribution, mechanical ventilation status, sample origin, and incidence of intraventricular hemorrhage. Values are expressed as mean ± standard deviation (sd) for continuous variables and number (percentage) for categorical variables.

### 3.2 Differentially Regulated Metabolites in Systemic Inflammation and Sepsis

The univariate investigation of differential metabolic abundances on the comprehensive data set revealed significant disruptions in amino acid and lipid metabolism across both the Inflamed and Sepsis models, underscoring the profound impact of systemic inflammation on the neonatal metabolome. There were 91 metabolites statistically significant in the Inflamed model (Figure 2a), with 30 metabolites (86.67% signaling lipids) elevated and 61 metabolites (57.38% amines) reduced in the inflamed group (Table S3). In the Sepsis model, 96 metabolites showed significant changes (Figure 2b), with an increase in 37 metabolites (81.08% signaling lipids) and a decrease in 59 metabolites (59.32% amines) in the sepsis group (Table S4). Results from C_SINS, C_S, and SINS_S models are provided in Figure S2 and Tables S5–S7.

**Figure 2:**
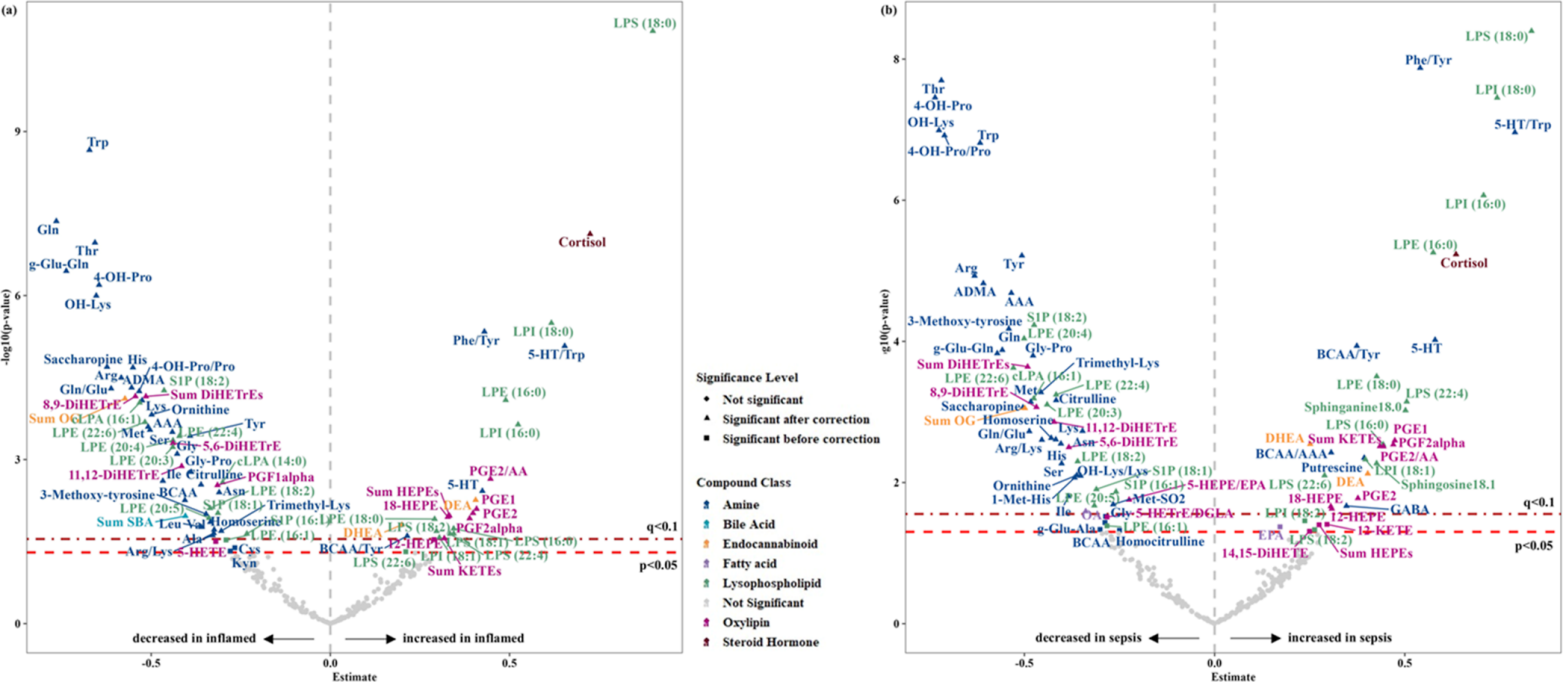
Differentially regulated metabolites in the Inflamed and Sepsis models. The figure presents volcano plots for the (a) Inflamed and (b) Sepsis models, showcasing the differential expression of metabolites by depicting the relationship between the estimate (*x*-axis) and statistical significance (−log10 *p*-value, *y*-axis). Metabolites are color-coded by compound classes, including amines, bile acids, endocannabinoids, fatty acids, lysophospholipids, oxylipins, and steroid hormones. Data points are shaped based on significance levels: significant after FDR correction, significant before FDR correction, and not significant. The vertical dashed line indicates an estimate of zero effect, and horizontal lines denote significance thresholds. FDR = false discovery rate.

Figure 3 illustrates a comprehensive comparison of key metabolite changes across all five models. ANOVA and fold change (FC) analyses further revealed distinct metabolic patterns across control, SINS, and sepsis groups.

**Figure 3:**
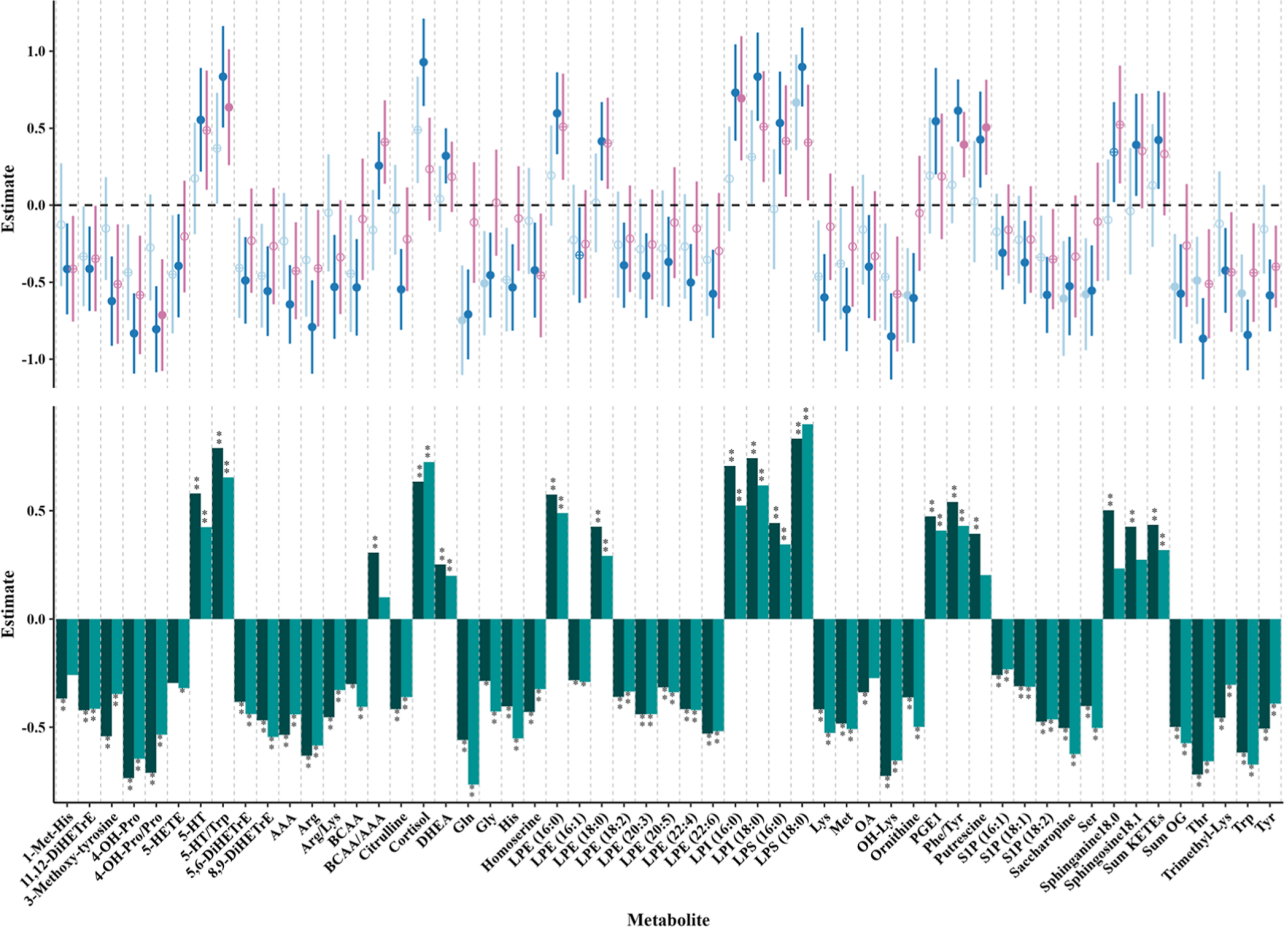
Comparative assessment of metabolic changes in inflammatory and septic conditions. The figure presents a comprehensive analysis of metabolite estimates derived from different linear mixed-effect models. The bottom panel shows bar plots comparing the estimates from the Inflamed (green) and Sepsis (dark green) model for each metabolite. The top panel features a forest plot displaying the estimates and confidence intervals of the additional models: C_SINS (light blue), C_S (blue) and SINS_S (pink). The dual-panel visualization allows for a comprehensive comparison of how metabolites are differentially regulated across these models, highlighting the similarities and differences in metabolite behavior in response to systemic inflammation-no sepsis and sepsis. Metabolite significance before and after FDR correction is depicted with different shapes: significant after correction (filled circle or **), significant before correction (circle with a plus or *), and not significant (circle or nothing). C_SINS = control versus systemic inflammation-no sepsis; C_S = control versus sepsis; SINS_S = systemic inflammation-no sepsis versus sepsis; FDR = False Discovery Rate.

#### 3.2.1 Etiology-Independent Metabolic Signatures of Systemic Inflammation

Among the 79 metabolites common between the Inflamed and Sepsis models, 30 demonstrated highly comparable levels with similar FC in both SINS and sepsis groups compared to the control (Figure 4a, Table S8). Notably, amino acids, including glycine, glutamine, lysine, histidine, serine, saccharopine, ornithine, and BCAA were consistently downregulated in both C_SINS and C_S models, with FC ranging from 0.72 to 0.91 for SINS and 0.74–0.90 for sepsis, indicating uniform trends across both inflammatory conditions. Similarly, arachidonic acid derivatives (5,6-DiHETrE, 8,9-DiHETrE, 11,12-DiHETrE, 5-HETE; FC: 0.55–0.77), monounsaturated sphingosine-1-phosphates (S1P(16:1), S1P(18:1); FC: 0.73–0.80), and unsaturated LPEs (LPE­(16:1), LPE(18:2), LPE(20:3), LPE(20:5), LPE(22:4), LPE(22:6); FC: 0.68–0.89) exhibited decreased levels in both inflammatory groups. However, the regulation of S1Ps and LPEs in C_SINS model did not reach the conventional threshold of *p* < 0.05, likely due to the smaller sample size and greater variability within the SINS group.

**Figure 4:**
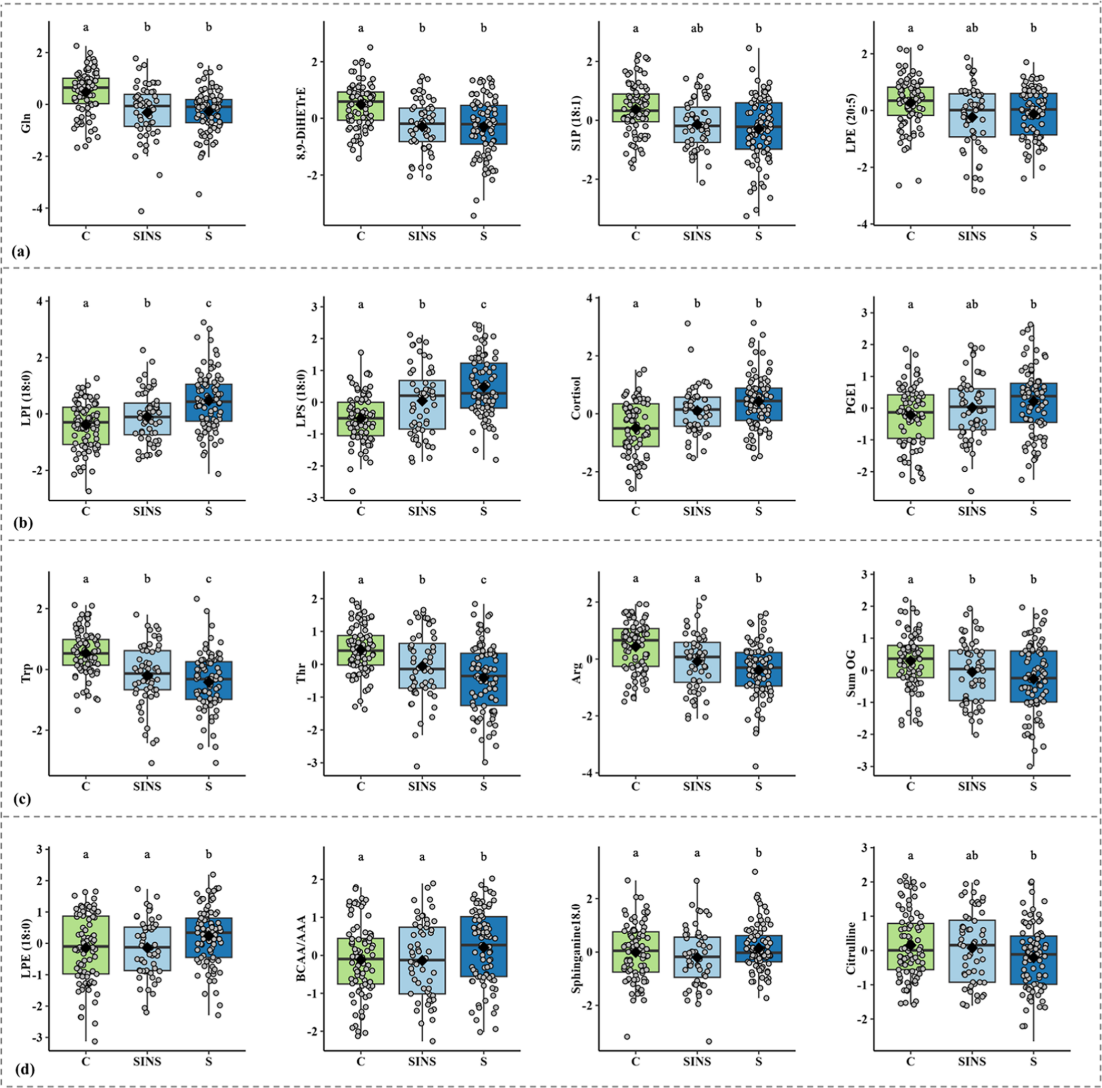
Metabolite level trends across control, systemic inflammation-no sepsis, and sepsis groups. The figure presents a series of boxplots illustrating the distribution of metabolite levels prior to confounder correction, highlighting specific trends across the three groups: (a) metabolites with similar levels in both SINS and sepsis compared to control, (b) metabolites exhibiting an increasing trend from control to SINS to sepsis, (c) metabolites showing a decreasing trend from control to SINS to sepsis, and (d) metabolites specifically upregulated or downregulated only in sepsis, with similar levels in control and SINS. In each boxplot, the central line represents the median, with box edges denoting the interquartile range. Individual data points represent samples, and the black diamond represents the mean. The letters denote significance between groups from the linear-mixed effect models: shared letters indicate no significant difference, while unique letters indicate significant difference. C = Control (green); SINS = Systemic Inflammation-No Sepsis (light blue); S = Sepsis (blue).

#### 3.2.2 Metabolic Markers of Enhanced Immune Dysregulation in Sepsis

Several metabolites demonstrated a progressive increase (Figure 4b) or decrease (Figure 4c) in their levels from control to SINS to sepsis (Table S8). Notably, LPS(18:0) and LPI(18:1) showed a consistent upward trend with increasing severity of inflammation, while tryptophan, threonine, hydroxylysine, 4-hydroxyproline and S1P(18:2) exhibited a consistent downward trend, with *p* < 0.05 in both C_SINS and SINS_S models. Additionally, cortisol (C_SINS FC: 2.4; SINS_S FC: 1.5), arginine (C_SINS FC: 0.79; SINS_S FC: 0.84) and sum of OG (C_SINS FC: 0.85; SINS_S FC: 0.92) displayed larger fold changes from control to SINS than SINS to sepsis. Alternatively, fold changes in tyrosine (C_SINS FC: 0.87; SINS_S FC: 0.79), 3-methoxy-tyrosine (C_SINS FC: 0.91; SINS_S FC: 0.82), AAA (C_SINS FC: 0.91; SINS_S FC: 0.85), and 4-hydroxyproline/proline (C_SINS FC: 0.88; SINS_S FC: 0.84) were higher in SINS to sepsis. Furthermore, PGE1 and arginine/lysine showed an increasing and decreasing trend, respectively, but achieved significance of *p* < 0.05 only in the extreme comparison of control to sepsis, as opposed to their subtle gradual shifts from control to SINS or SINS to sepsis.

#### 3.2.3 Sepsis-Specific and SINS-Specific Metabolic Dysregulations

In the analysis of the Inflamed model, several metabolites showed statistical significance driven exclusively by the sepsis group, with similar levels in control and SINS but distinct differential regulation in sepsis, suggesting their potential as sepsis-specific biomarkers (Table S8, Figure 4d). Notably, serotonin (FC: 2.27), saturated lysophospholipids (LPE(16:0), LPE(18:0), LPI(16:0), LPS(16:0); FC: 1.04–1.35), DHEA (FC: 1.12), and sum of KETEs (FC: 1.32) were elevated, while citrulline (FC: 0.83), homoserine (FC: 0.89), and trimethyl lysine (FC: 0.95), were decreased in sepsis. Additional sepsis-specific signatures identified from the Sepsis model included increased levels of putrescine (FC: 1.69), sphinganine(18:0) (FC: 1.04), sphingosine(18:1) (FC: 1.00), and BCAA/AAA (FC: 1.13), alongside reductions in oleic acid (FC: 0.95) and 1-methyl-histidine (FC: 0.96). However, the association of LPS(16:0), trimethyl-lysine, sphinganine(18:0), sphingosine(18:1), oleic acid and 1-methyl-histidine with sepsis became significant only after correction for confounders.

PGF1alpha was observed to be significantly downregulated exclusively in the SINS group when compared to the controls, underscoring its potential as a SINS-specific biomarker (Figure S3a). Additionally, when stratifying the sepsis group by blood culture outcome, PGF1alpha was significantly downregulated in culture-negative sepsis, whereas culture-positive sepsis exhibited an upward trend (Figure S3b, Table S9).

### 3.3 Sex-Specific Phenotypic Distinction in Inflammatory Response to Sepsis

Sex stratification on the comprehensive data set resulted in a male cohort (*n* = 54, 136 samples) with 55 sepsis samples (40.44%), and a female cohort (*n* = 30, 91 samples) with 30 sepsis samples (32.97%). The sex-stratified Sepsis models identified a pro-inflammatory phenotype in males, characterized by elevated prostaglandins (PGE1 (Estimate = 0.46), PGE2 (Estimate = 0.47), PGF2alpha (Estimate = 0.61), delta-17,6-keto-PGF1alpha (Estimate = 0.52)), and decreased DiHETrEs (Estimate = −0.49 to −0.69) and sum of OG (Estimate = −0.70). Additionally, increased levels of serotonin (Estimate = 0.82), GABA (Estimate = 0.45), sphinganine(18:0) (Estimate = 0.55), and sphingosine(18:1) (Estimate = 0.45), as well as decreased levels of trimethyl lysine (Estimate = −0.55), and homoserine (Estimate = −0.46) were found to be exclusively and significantly associated with sepsis in males. These associations were identified only after confounder correction; therefore, raw FC are not reported. Females, on the other hand, exhibited an anti-inflammatory phenotype, characterized by increased omega-3 FAs and their metabolites, including EPA (Estimate = 0.35) and its derivatives (9-HEPE (Estimate = 0.42), 12-HEPE (Estimate = 0.52), 15-HEPE (Estimate = 0.52), 18-HEPE (Estimate = 0.54)), and DHA metabolites (16-HDoHE (Estimate = 0.38), 17-HDoHE (Estimate = 0.42), 20-HDoHE (Estimate = 0.47)). Increased levels of DHEA (Estimate = 0.43), sum of KETEs (Estimate = 0.64), and AEA (Estimate = 0.41), as well as decreased levels of citrulline (Estimate = −0.65) and histidine (Estimate = −0.71), were also found to be predominantly driven by females. Although some female-driven metabolites did not attain statistical significance of *q* < 0.1, likely due to the smaller cohort size, consistent trends within metabolite classes (*p* < 0.05) support these observed sex-specific differences. Figure 5a illustrates the sex-specific response to sepsis in the two genders with a more detailed comparison of metabolites including significance values and shared metabolites across sexes presented in Figure S4a and Table S10.

**Figure 5:**
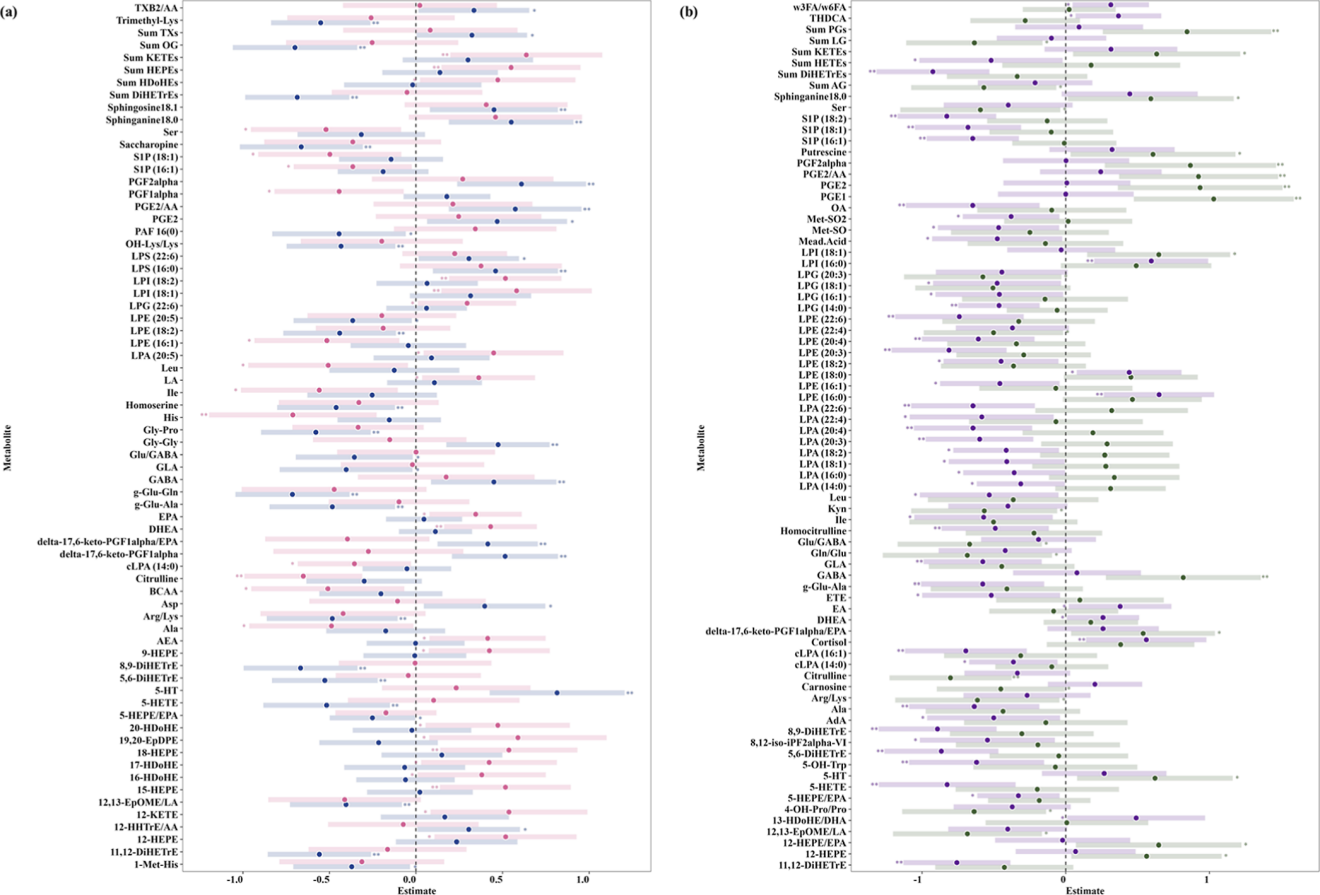
Forest plots of metabolite estimates in sex and pathogen-stratified sepsis models. The figure presents forest plots illustrating the estimates with significance, before and after FDR correction, of metabolite levels in linear-mixed effect sepsis models stratified by sex and pathogen type. Confidence intervals are represented by the horizontal bars. The left panel (a) shows the estimates for male (dark blue) and female (pink) sepsis models, while the right panel (b) displays the estimates for gram-positive (purple) and gram-negative (dark green) sepsis models. These plots highlight the differences in metabolite regulation influenced by sex and pathogen type, providing a comprehensive comparison of the metabolic responses across these stratified groups. FDR = False Discovery Rate; Significance after correction = “**”; Significance before correction = “*”; Not significant = “ ”.

### 3.4 Pathogen-Driven Metabolomic Heterogeneity in Sepsis

Of the 43 culture-positive sepsis samples in the comprehensive data set, 29 were associated with gram-positive bacteria and 14 with gram-negative bacteria, with similar male-to-female ratio, indicating no sex bias in the stratification analysis (Fisher’s exact test *p* = 1). Pathogen-stratification in the Sepsis models elucidated metabolic dysregulations uniquely associated with each pathogen type (Figure 5b). An extensive comparison of metabolite profiles, including *p*-values and shared metabolites across both bacterial groups, is provided in Figure S4b and Table S11. Patients with gram-negative sepsis exhibited significantly increased levels of COX enzyme induced metabolites (PGE1 (Estimate = 1.03), PGE2 (Estimate = 0.94), PGF2alpha (Estimate = 0.87)) and reduced levels of endocannabinoids (sum of OG (Estimate = −0.93), sum of LG (Estimate = −0.64)) and citrulline (Estimate = −0.80). Further, serotonin (Estimate = 0.62), putrescine (Estimate = 0.61), sum of KETEs (Estimate = 0.63), 4-hydroxyproline/proline (Estimate = −0.64), and arginine/lysine (Estimate = −0.62) were found to be predominantly driven by gram-negative sepsis with *p* < 0.05 (*q* > 0.1). On the contrary, gram-positive sepsis was characterized by decreased levels of CYP enzyme induced DiHETrEs (Estimate = −0.65 to −0.89), unsaturated lysophospholipids, including LPEs (Estimate = −0.45 to −0.81) and LPAs (Estimate = −0.41 to −0.65), and increased levels of cortisol (Estimate = 0.56), DHEA (Estimate = 0.26), and omega-3/omega-6 FA ratio (Estimate = 0.31). Some LPEs and LPAs, DHEA, and omega-3/omega-6 FA ratio did not achieve the statistical significance of *q* < 0.1.

### 3.5 Diagnostic Potential of Metabolites for Early and Accurate Detection of Sepsis

The logistic regression models identified 26 significant metabolites (*q* < 0.1) in the C_S model, while no significant hits were observed in the C_SINS and SINS_S models, likely due to the limited sample size in the STP data set. A comparison of metabolites with *p* < 0.05 between the C_S and C_SINS models revealed that 31 of the 61 metabolites were exclusively associated with sepsis (Figure S5a), with a substantial overlap observed with LMM results. Additionally, analysis of the SINS_S model identified metabolites such as cortisol, putrescine, 11beta-PGF2alpha/AA, S1P(18:2), and LPA(20.4) as potential biomarkers for distinguishing SINS from sepsis (Figure S5b). Details of these results are documented in Supporting Information S5 and Tables S12–S14.

The predictive performance of the metabolomic diagnostic framework for distinguishing SINS from sepsis at the point of clinical suspicion, as estimated using bootstrap-aggregated LASSO logistic regression across 100 iterations, yielded a mean AUC of 0.61, sensitivity of 0.69 and specificity of 0.66. Detailed performance metrics for each iteration are provided in Table S15. Feature selection stability analysis identified five metabolites, including 11-beta-PGF2alpha, o-acetyl-serine, PGE1, 8-iso-PGF3alpha, and taurine, which were consistently selected in over 45% of the bootstrap models. The multivariable logistic regression model constructed using these 5 metabolites demonstrated a good discriminatory performance, with an AUC of 0.75, sensitivity of 0.82, specificity of 0.73, PPV of 0.9, and F1 score of 0.86. In contrast, models based on individual inflammatory markers showed limited diagnostic utility (AUCs: CRP = 0.53, IL-6 = 0.59, PCT = 0.55), and their combination further reduced performance (AUC:0.49). However, integration of the metabolite panel to the individual inflammatory markers led to improved classification. The highest diagnostic performance was achieved by combining the metabolite panel with IL-6 (McNemar *p*-value: 0.01), resulting in an AUC of 0.79, sensitivity of 0.85, specificity of 0.82, PPV of 0.93, and F1 score of 0.89. Combinations with either CRP (McNemar *p*-value: 0.02) or PCT (McNemar *p*-value: 0.002) yielded an AUC of 0.74. Receiver operating characteristic curve and model parameters are provided in Figure 6 and Table S16.

**Figure 6:**
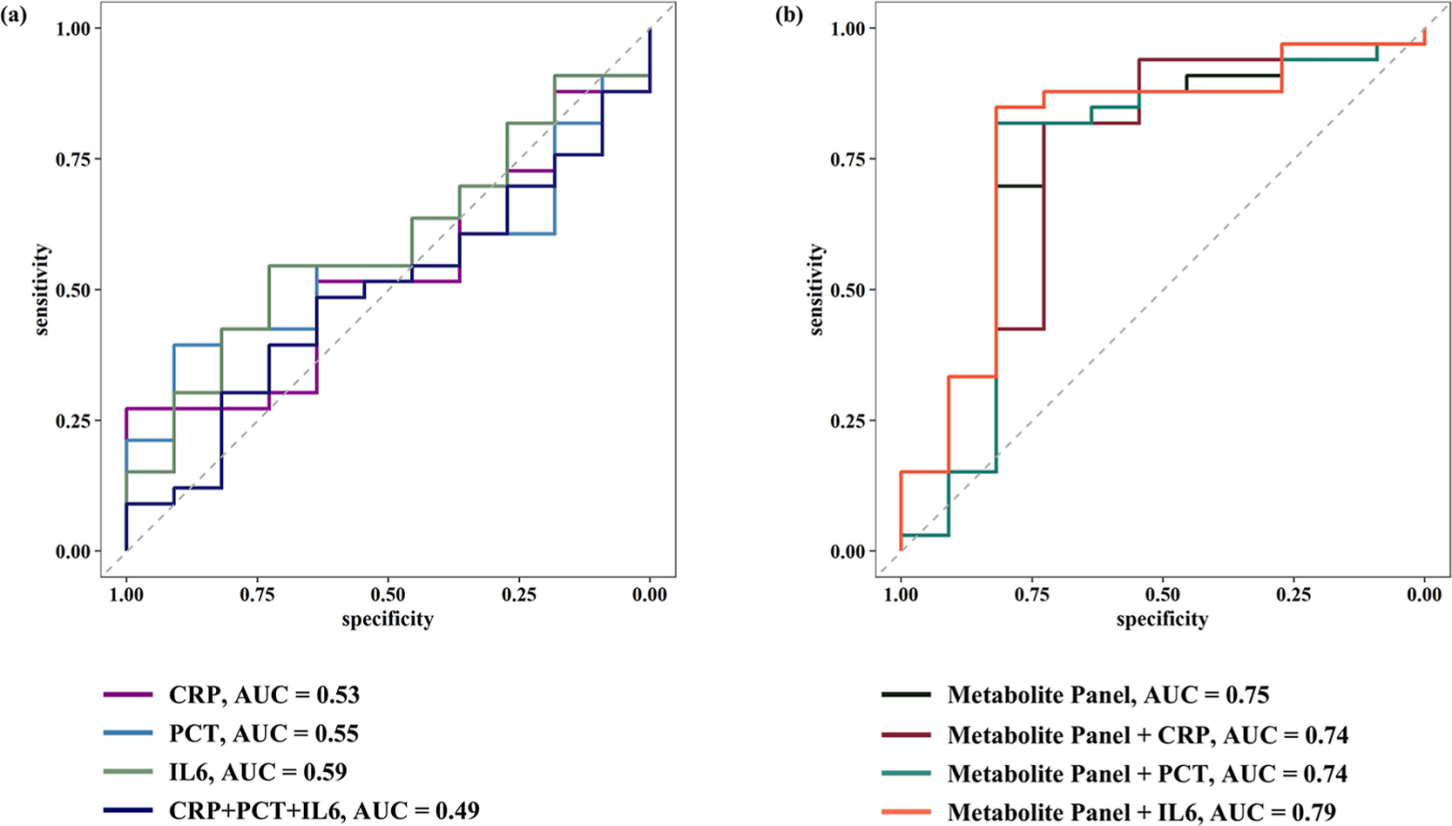
Area under the receiver operating characteristic curves for diagnostic models differentiating systemic inflammation-no sepsis from sepsis at moment of suspicion. The figure presents the receiver operating characteristic curves with the area under the curve for (a) individual and combined routine inflammatory marker models and (b) metabolomic and integrated models. The metabolite panel includes 5 metabolites: 11beta-PGF2alpha, o-acetyl serine, PGE1, 8-iso-PGF3alpha, and taurine. The area under the curve values obtained from the leave-one-out cross-validation demonstrates the diagnostic performance of these models in distinguishing between systemic inflammation-no sepsis and sepsis at the moment of suspicion. Spearman correlation analyses revealed differential correlations between diagnostic panel metabolites and inflammatory markers in SINS and sepsis, as illustrated in Figure S6. Notably, o-acetyl-serine displayed a strong positive correlation with CRP (ρ_s_ = 0.66, *p* = 0.03) and PCT (ρ_s_ = 0.71, *p* = 0.01) in SINS, whereas no correlation was observed in sepsis (CRP: ρ_s_ = −0.01, *p* = 0.94; PCT: ρ_s_ = −0.24, *p* = 0.19). Similarly, 8-iso-PGF3alpha (ρ_s_ = −0.76, *p* = 0.0092) exhibited a negative correlation with IL-6 in SINS, but no correlation in sepsis (ρ_s_ = −0.1, *p* = 0.58).

## 4 Discussion

To the best of our knowledge, this is the first metabolomic study conducted on preterm neonates that delineates the similarities and differences between SINS and sepsis using well-defined patient subgroups for analysis. The study further explores the potential of metabolomics to unravel the observed clinical disparities in sex-specific and pathogen-specific sepsis presentations, facilitating potential personalized treatment strategies. A comprehensive overview of the key findings in the study summarizing the results across all models, is shown in Figure 7.

**Figure 7:**
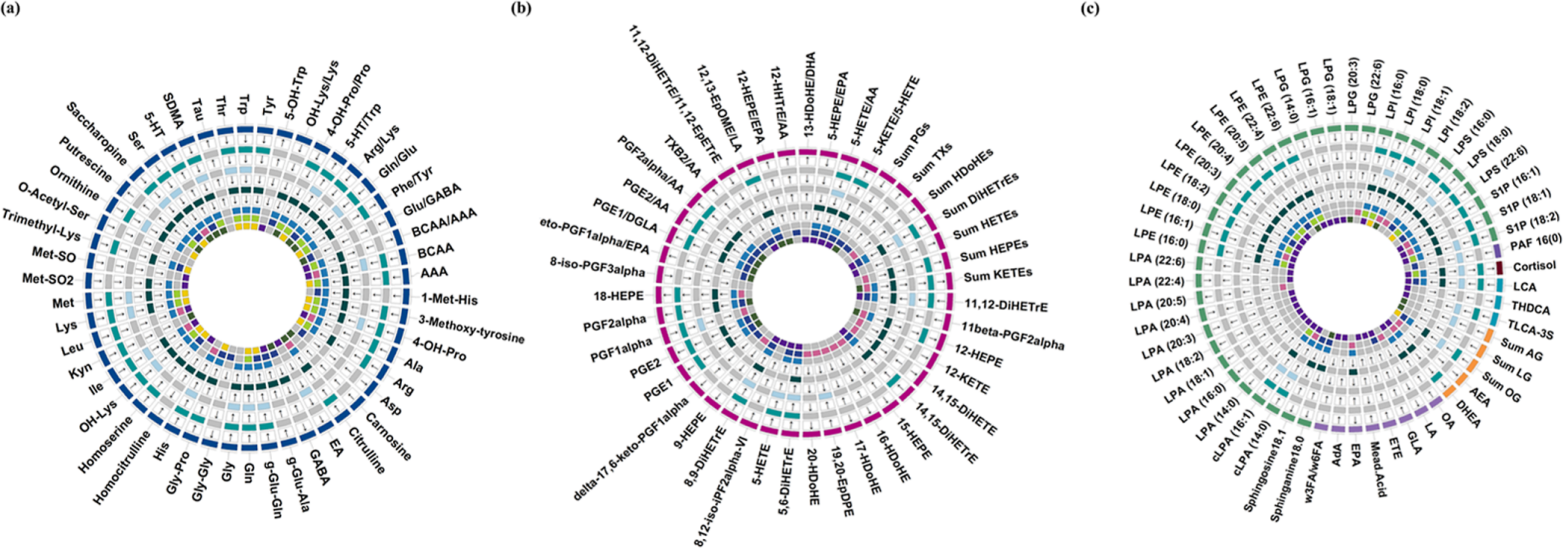
Comprehensive summary of compound classification and statistical associations across models. Circos plots summarize the direction and significance of metabolite associations across various linear mixed-effect models for (a) amines, (b) oxylipins, and (c) lysophospholipids, endocannabinoids, fatty acids, steroid hormone, and platelet activating factor. Each plot comprises 11 concentric tracks representing compound classification and statistical associations. From the outermost track, track 1 represents the compound class: oxylipins (dark pink), platelet activating factor (dark purple), steroid (dark brown), amines (dark blue), bile acids (light blue), endocannabinoids (yellow), fatty acids (light purple), and lysophospholipids (green). Track 2 and Track 3 represent the direction of estimate and significance (*p* < 0.05: green, *p* > 0.05: gray) in the Inflamed model. Track 4 and Track 5 display the direction of estimate and significance (*p* < 0.05: light blue, *p* > 0.05: gray) in the C_SINS model. Track 6 and Track 7 show the direction of estimate and significance (*p* < 0.05: dark green, *p* > 0.05: gray) in the Sepsis model. Track 8 and Track 9 represent the direction of estimate and significance (*p* < 0.05: blue, *p* > 0.05: gray) in the C_S model. Track 10 represents the significance in gender-stratified sepsis models (*p* < 0.05: only in males (blue), only in females (pink), in both males and females (green). Track 11 represents the significance in pathogen-stratified sepsis models (*p* < 0.05: only in gram-positive sepsis (dark purple), only in gram-negative sepsis (dark green), in both gram-positive and gram-negative sepsis (gold). Arrows are interpreted from the perspective of the center of the plot. C_SINS = control vs systemic inflammation-no sepsis; C_S: control vs sepsis.

The uniform downregulation of several amino acids and signaling lipids in SINS and sepsis reflects the body’s fundamental response mechanism to systemic inflammation, regardless of the causative trigger. These findings underscore the tremendous metabolic stress experienced by the infants, driven by increased metabolic demands and disruptions in synthesis or recycling pathways. Reduced amino acid levels suggest impairments in protein synthesis and metabolism, essential for maintaining immune function and cellular repair.^22,23^ The significant decrease in glutamine may be attributed to its heightened utilization by proliferating immune cells, a hallmark of systemic inflammation.^22^ Similarly, reduced BCAAs, key activators of the mTORC1 pathway for protein synthesis, indicate a shift toward protein catabolism, often linked to muscle wasting in inflammatory conditions.^23,24^ Further, reduced levels of unsaturated LPEs and histidine indicate oxidative stress and redox imbalance, potentially driven by reactive oxygen species oxidizing the LPE double bonds and depleting antioxidant reserves.^25^ The downregulation of metabolites with anti-inflammatory properties, such as glycine,^26^ serine,^27^ and DiHETrEs,^28^ reflects an inadequate compensatory response to heightened pro-inflammation. The depletion of lysine and glycine, both essential for collagen production, indicates impaired tissue repair capacity.^29,30^ These uniform metabolic perturbations underscore the challenge in differentiating systemic inflammation arising from infectious vs non-infectious causes and offer promising targets for restoring metabolic balance and improving patient outcomes in systemic inflammation.

Metabolites exhibiting progressive trends from control to SINS to sepsis deepen the understanding of pathways associated with escalating inflammatory burden. The increasing severity of inflammation necessitates higher production of acute phase proteins, imposing a considerable metabolic strain reflected in, among others, the declining levels of threonine, which is essential for acute-phase response and gut barrier integrity.^31^ The dysregulation of S1Ps, particularly declining S1P(18:2) levels which are lowest in sepsis, further compromises intestinal and endothelial integrity, promoting vascular permeability, leakage, and edema that contribute to sepsis-induced organ failure.^32^ Inhibition of S1P degradation has shown tissue-protective effects in sepsis, reducing severity and improving survival by enhancing disease tolerance.^33^ The declining arginine levels, attributed to increased nitric oxide production and reduced *de novo* synthesis as widely reported in sepsis studies, may impair vascular function, neurotransmission, and host defenses.^34–37^ Arginine insufficiency has been hypothesized as a key factor impairing immune responses in preterm neonates during infections.^38^ High cortisol levels promote pro-inflammatory cytokine production thus exacerbating inflammation in sepsis.^39^ While arginine and cortisol changes primarily reflect inflammatory responses, metabolites such as tyrosine and 4-hydroxyproline/proline demonstrate more pronounced changes in response to infection. Decreased tyrosine levels in sepsis may result from increased utilization for catecholamine synthesis or impaired conversion from phenylalanine due to enzymatic dysfunction.^40,41^ Further, the lower 4-hydroxyproline/proline ratio reflects higher tissue damage in sepsis, as 4-hydroxyproline is essential for collagen stability.^42^ Overall, these trends highlight the greater severity and deterioration in sepsis compared to SINS, offering insights into potential diagnostic thresholds and therapeutic targets.

Exclusively altered metabolites in the sepsis group provide crucial insights into sepsis pathophysiology and potential diagnostic biomarkers. For instance, elevated serotonin levels have been linked to several sepsis-associated complications ranging from enhancing bacterial virulence to fostering a pro-thrombotic environment leading to ischemia and organ failure.^43–45^ Exacerbated depletion of platelets and coagulation factors has been identified as an independent risk factor for neonatal sepsis-associated mortality.^46^ Elevated saturated lysoglycerophospholipids in sepsis reflect heightened phospholipase A2 enzyme activity, which hydrolyzes membrane phospholipids to produce lysoglycerophospholipids and free FAs.^47^ These saturated lipid species accumulate under oxidative stress, likely due to their enhanced stability and resistance to peroxidation from their lack of double bonds. Elevated putrescine, driven by enhanced ornithine decarboxylase enzyme activity, indicates the body’s attempt to regulate immune function and cellular repair in sepsis.^48,49^ This shift in ornithine metabolism reduces citrulline levels, reflecting the impaired *de novo* arginine and nitric oxide production.^36^ Similarly, higher sphinganine(18:0) in sepsis represents a protective mechanism against intestinal barrier injury.^50^ Furthermore, the significant downregulation of PGF1alpha in SINS, mirrored in culture-negative sepsis group and contrasted by an opposite trend in culture-positive sepsis, suggests that the presence of detectable pathogens may distinctly influence PGF1alpha regulation. The absence of detectable pathogens in culture-negative sepsis cases raises the possibility of misclassified SINS, potentially driving the observed downregulation in the culture-negative sepsis group. Therefore, PGF1alpha could be a potential marker to distinguish between SINS and true sepsis cases in the absence of detectable pathogens. Collectively, these metabolic changes illustrate the unique and complex adaptive responses in sepsis, highlighting the differences between SINS and sepsis.

Sex-stratified analysis identified metabolites significantly associated with sepsis in either male or female preterm neonates, suggesting potential sex-specific immune responses. In males, the decreased levels of anti-inflammatory DiHETrEs and OGs, coupled with elevated pro-inflammatory prostaglandins such as PGE2 and PGF2alpha, suggest a skewed inflammatory balance toward a heightened pro-inflammatory state. These arachidonic acid-derived eicosanoids are well-established mediators of inflammation and have been linked to severe inflammatory responses.^51^ Elevated serotonin levels observed in males may further exacerbate this pro-inflammatory milieu by promoting platelet activation and thrombus formation, which is consistent with clinical data showing a greater predisposition of males to severe sepsis outcomes and complications, including disseminated intravascular coagulation. Meanwhile, females exhibited increased levels of w3-FAs EPA and DHA, their metabolites (HEPEs, HDoHEs), and ethanolamine endocannabinoids, which are known to resolve inflammation, modulate immune cell activity, and maintain homeostasis.^52^ The enhanced production of these metabolites, potentially driven by hormonal or genetic factors, may reflect a protective mechanism that mitigates the severity of sepsis, explaining the more robust and controlled inflammatory response and better outcomes observed in female neonates.^53–55^ While these observations align with reported sexual dimorphism in infectious diseases, interaction testing between sexes were not performed due to limited sample size.^56^ Nonetheless, our sex-stratified models highlight biologically plausible, sex-specific metabolic associations that may contribute to differences in sepsis susceptibility and outcomes, and offer a foundation for future studies exploring sex-informed diagnostic or therapeutic strategies.

Pathogen-stratified analysis identified metabolites significantly associated with sepsis in either gram-positive or gram-negative bacterial infections, suggesting distinct metabolic responses to different causative pathogens. In gram-negative sepsis, elevated levels of COX enzyme-induced metabolites such as PGE2 and PGF2alpha were observed, consistent with a previous study reporting stronger PGE2 production in human monocytes in response to gram-negative bacteria than gram-positive bacteria.^57^ The concurrent reduction in glycerol endocannabinoids suggest a disrupted endocannabinoid system which is crucial for immune regulation.^58^ Elevated levels of serotonin, putrescine, and sum of KETEs further reflect severe clinical outcomes and tissue damage. Meanwhile, gram-positive sepsis was characterized by decreased CYP enzyme-induced metabolites like DiHETrEs and unsaturated LPEs and LPAs, suggesting disrupted FA metabolism and signaling.^59^ Interestingly, increased levels of DHEA and w3FA/w6FA ratio were observed, potentially reflecting adaptive immune regulatory responses in gram-positive sepsis. These differences between gram-positive and gram-negative bacterial sepsis may arise from the rapid clinical presentation of the latter, while the subtler symptoms of the former often delay diagnosis. Alternatively, these differences may reflect distinct immune signaling mechanisms, as lipopolysaccharides from gram-negative bacterial outer membrane activate toll-like receptor 4 (TLR4), triggering potent immune responses, while the peptidoglycan cell wall of gram-positive bacteria predominantly interact with TLR2/TLR6 heterodimer.^60^ These findings suggest pathogen-specific metabolic profiles, which may serve as potential biomarkers for early identification of pathogens or tailored therapeutic targets for effective and personalized interventions, reducing unnecessary broad-spectrum antibiotic use.

The multivariable model comprising 11-beta-PGF2alpha, o-acetyl-serine, PGE1, 8-iso-PGF3alpha, and taurine demonstrated good sensitivity and specificity in distinguishing SINS from sepsis at the moment of suspicion. In contrast, inflammatory marker models showed limited diagnostic utility, underscoring the inadequacy of relying solely on routine clinical biomarkers, which aligns with clinical observations where blood cultures and follow-up samples are required for accurate diagnosis. The integration of inflammatory markers with the metabolic panel significantly improved diagnostic performance, emphasizing the synergistic value of combining diverse biomarker types. The model integrating the metabolic panel with IL-6 and achieving an AUC of 0.79, with a sensitivity of 0.85 and a specificity of 0.82, reflect both robust detection of true sepsis cases and effective reduction of false positives. This balanced diagnostic profile enhances clinical utility by supporting timely recognition of sepsis while minimizing unnecessary treatment. When used in conjunction with clinical assessment, such an integrated biomarker approach holds promise for improving diagnostic precision in neonatal sepsis and guiding more targeted therapeutic interventions.

The results presented in the study align with known biological mechanisms and clinical observations, but caution is advised, as validation in a larger, more balanced cohort is necessary. However, obtaining an appropriately sized validation cohort may be challenging as preterm neonates are a particularly vulnerable population. Despite the comprehensive data set having a good sample size, the power is reduced in the STP data set and the unbalanced data sets created for the stratified analyses. The stratification analyses were thus performed to elucidate metabolic changes rather than identify diagnostic biomarkers for sex- or pathogen-specific treatments. However, the strong differences between the groups suggest potential diagnostic utility and avenues for targeted treatments upon further validation. Additionally, it is crucial to investigate whether sex-specific or universal biomarkers offer the best approach for diagnosing sepsis in both sexes. The ambiguity surrounding culture-negative sepsis introduces heterogeneity to the sepsis group. This complexity underscores the importance of validation to delineate true sepsis-related metabolic alterations from other inflammatory conditions. Future work should focus on validation of the multivariable model to accurately assess diagnostic accuracy, reproducibility, and clinical utility in larger cohorts over multiple centers. Additionally, these findings need to be translated into routine clinical practice which requires robust, portable, user-friendly, and cost-effective devices capable of rapid and accurate metabolite quantification. With continued collaboration between academia, technology developers, clinicians, and regulatory bodies, the findings of this study hold significant potential in paving the way for bedside metabolomics-based diagnostics.

## 5 Conclusions

In conclusion, this study provides a pivotal advancement in the understanding of late-onset sepsis in preterm neonates, with the potential to fundamentally alter the current diagnostic and treatment landscape. The application of metabolomics to distinguish between SINS and sepsis at the moment of suspicion is a critical breakthrough. Accurate identification of SINS could enable clinicians to align treatment decisions with the actual inflammatory status of the neonate, reserving antibiotics for confirmed infections and mitigating risks such as antibiotic resistance, adverse drug reactions, and microbiome disruption. The sex- and pathogen-specific insights from this study further highlight the potential for targeted therapies, including the use of narrow-spectrum antibiotics to enhance treatment precision and efficacy, marking a substantial improvement over the current reliance on empirical broad-spectrum antibiotic use. Personalized interventions based on the neonate’s metabolic profile could improve outcomes while reducing the limitations of a one-size-fits-all strategy. Additionally, these findings could help clinicians predict which infants are at a greater risk of severe outcomes, allowing for closer monitoring and earlier intervention in high-risk cases. Clinically, integrating metabolomics into routine diagnostics could result in the development of rapid, bedside tests that provide actionable insights within hours. Ultimately, this metabolomics-driven approach could reshape the future of neonatal sepsis management, offering a new standard of care that enhances survival, reduces antibiotic misuse, and provides preterm neonates with a better chance at a healthy start in life.

## Supplementary Material





## Data Availability

All processed metabolomics data (peak area ratios derived from SCIEX software) and curated metadata are available in a publicly accessible GitHub repository: Manchu1205/MOMETA-Study. The repository also contains the complete analysis pipeline, including preprocessing scripts, statistical modeling code, and figure generation workflows, to ensure full transparency and reproducibility of the findings. In addition, raw LC–MS files and associated metadata have also been deposited to the MetaboLights repository under the study accession number MTBLS10665 (www.ebi.ac.uk/metabolights/MTBLS10665).

## Notes

S1: cohort group definitions and sample processing; S2: metabolomic data acquisition; S3: data preprocessing; S4: statistics; S5: univariate logistic regression model results; Figure S1: temporal distribution of samples in the comprehensive and single time point data sets; Figure S2: differentially regulated metabolites in the C_SINS, C_S, and SINS_S models; Figure S3: differential regulation of PGF1alpha across SINS and sepsis subgroups; Figure S4: comparative analysis of differentially regulated metabolites in sex- and pathogen-stratified linear mixed-effect sepsis models; Figure S5: diagnostic potential of metabolites derived from univariate logistic regression analysis; Figure S6: spearman correlation analyses of the metabolites in the diagnostic panel with the routine inflammatory markers (PDF)
Table S1: List of included metabolic features and their abbreviations; Table S2: Missing data patterns of metabolites exhibiting greater than 20% missingness; Table S3: Linear mixed-effect model results of the “Inflamed” model comparing control with inflamed groups; Table S4: Linear mixed-effect model results of the “Sepsis” model comparing nonsepsis groups with sepsis; Table S5: Linear mixed-effect model results of the “C_SINS” model comparing control with systemic inflammation-no sepsis; Table S6: Linear mixed-effect model results of the “C_S” model comparing control with sepsis; Table S7: Linear mixed-effect model results of the “SINS_S” model comparing systemic inflammation-no sepsis with sepsis; Table S8: raw fold change values and corresponding linear mixed-effect model *p*-values of highlighted metabolites; Table S9: linear mixed-effect model results of the blood culture-stratified “Sepsis” models; Table S10: linear mixed-effect model results of the gender-stratified “Sepsis” models; Table S11: linear mixed-effect model results of the pathogen-stratified “Sepsis” models; Table S12: logistic regression results of the “C_SINS” model comparing control with systemic inflammation-no sepsis in the single time point data set; Table S13: logistic regression results of the “C_S” model comparing control with sepsis in the single time point data set; Table S14: logistic regression results of the “SINS_S” model comparing systemic inflammation-no sepsis with sepsis in the single time point data set; Table S15: performance metrics of multivariable metabolomic models across 100 bootstrap iterations; Table S16: model parameters of diagnostic models to differentiate between systemic inflammation-no sepsis and sepsis at the moment of suspicion (XLSX)

## Abbreviations

AAA: aromatic amino acids
ANOVA: analysis of variance
AUC: area under the curve
BCAA: branched chain amino acids
COX: cyclooxygenase
CRP: C-reactive protein
CYP: cytochrome P
FA: fatty acid
FC: fold change
IL-6: interleukin-6
LASSO: least absolute shrinkage and selection operator
LC: liquid chromatography
LMM: linear mixed model
LOS: late-onset sepsis
MS: mass spectrometry
mTORC1: mammalian target of rapamycin complex 1
PCA: principal component analysis
PCT: procalcitonin
PPV: positive predictive value
SINS: systemic inflammation-no sepsis
STP: single time point
TLR: toll-like receptor
