# Supplementary material for: Survival of the Littlest: Navigating Sepsis Diagnosis beyond Inflammation in Preterm Neonates
Source: J Proteome Res. 2025 Apr 30;24(6):2846–60. doi: 10.1021/acs.jproteome.4c01072 (PMC12150309; doi:10.1021/acs.jproteome.4c01072)
Supplement: Supplementary file 1 [file pr4c01072_si_001.pdf]

Survival of the littlest: Navigating sepsis diagnosis beyond inflammation in preterm neonates

Manchu Umarani Thangavelu[1], Alida Kindt[1], Shawen Hassan[2], Jelte J.B. Geerlings[1], Charlotte Nijgh-van Kooij[1], Irwin K.M. Reiss[2], Bert Wouters[1], H. Rob Taal[2], and Thomas Hankemeier[1]

[1]Metabolomics and Analytics Center, Leiden University, Leiden, The Netherlands; [2]Department of Neonatal and Pediatric Intensive Care, Division of Neonatology, Erasmus MC, Rotterdam, The Netherlands

TABLE OF CONTENTS

S1: Cohort Group Definitions and Sample Processing

S2: Metabolomic Data Acquisition

S3: Data Preprocessing

S4: Statistics

S5: Univariate Logistic Regression Model Results

Figure S1: Temporal distribution of samples in the comprehensive and single time point datasets

Figure S2: Differentially regulated metabolites in the C_SINS, C_S, and SINS_S models

Figure S3: Differential regulation of PGF1alpha across SINS and sepsis subgroups

Figure S4: Comparative analysis of differentially regulated metabolites in sex- and pathogen-stratified linear mixed-effect sepsis models

Figure S5: Diagnostic potential of metabolites derived from univariate logistic regression analysis

Figure S6: Spearman correlation analyses of the metabolites in the diagnostic panel with the routine inflammatory markers

Table S1: List of included metabolic features and their abbreviations (xlsx)

Table S2: Missing data patterns of metabolites exhibiting greater than 20% missingness (xlsx)

Table S3: Linear mixed-effect model results of the "Inflamed" model comparing control with inflamed groups (xlsx)

Table S4: Linear mixed-effect model results of the "Sepsis" model comparing non-sepsis groups with sepsis (xlsx)

Table S5: Linear mixed-effect model results of the "C_SINS" model comparing control with systemic inflammation-no sepsis (xlsx)

Table S6: Linear mixed-effect model results of the "C_S" model comparing control with sepsis (xlsx)

Table S7: Linear mixed-effect model results of the "SINS_S" model comparing systemic inflammation-no sepsis with sepsis (xlsx)

Table S8: Raw fold change values and corresponding linear mixed-effect model p-values of highlighted metabolites (xlsx)

Table S9: Linear mixed-effect model results of the blood culture-stratified "Sepsis" models (xlsx)

Table S10: Linear mixed-effect model results of the gender-stratified "Sepsis" models (xlsx)

Table S11: Linear mixed-effect model results of the pathogen-stratified "Sepsis" models (xlsx)

Table S12: Logistic regression results of the "C_SINS" model comparing control with systemic inflammation-no sepsis in the single time point dataset (xlsx)

Table S13: Logistic regression results of the "C_S" model comparing control with sepsis in the single time point dataset (xlsx)

Table S14: Logistic regression results of the "SINS_S" model comparing systemic inflammation-no sepsis with sepsis in the single time point dataset (xlsx)

Table S15: Performance metrics of multivariable metabolomic models across 100 bootstrap iterations (xlsx)

Table S16: Model parameters of diagnostic models to differentiate between systemic inflammation-no sepsis and sepsis at the moment of suspicion (xlsx)

S1. Cohort Group Definitions and Sample Processing

- Culture-positive sepsis: samples with confirmed gram-positive or gram-negative bacteria in blood cultures.
- Culture-negative sepsis: samples where blood cultures were negative, yet sepsis symptoms persisted. These are defined by (a) CRP levels exceeding 10 mg/L within 2 days after blood culture, (b) administration of antibiotic therapy longer than 5 days (or intention to treat longer), and (c) clinical symptoms of sepsis assessed by the treating physician.
- No sepsis or SINS: samples from neonates initially displaying symptoms of sepsis with subsequent reassuring clinical status (no/mild symptoms) and negative blood cultures.

The sample collection process entailed acquiring 500 µL of lithium-heparin blood from neonates either through an arterial line or the heel prick technique. The blood sample was centrifuged at 3,000g for 5 min at room temperature to obtain plasma. A fraction of the isolated plasma was utilized at the discretion of clinical necessity for routine serological analysis, including measurements of clinical biomarkers of inflammation: CRP, IL-6, and PCT. Following the analysis, any residual plasma was initially stored at 4°C, and subsequently relocated to a long-term storage facility at -80°C within 24h for biobanking purposes. Upon sample selection, specific samples were retrieved from the biobank and transported to the analytical chemistry laboratory on dry ice. Upon arrival, these samples were stored at -80°C until aliquoting for metabolomic analysis.

S2. Metabolomic Data Acquisition

S2.1 Amines

Amino acids and biogenic amines in plasma were profiled using ultra performance liquid chromatography tandem mass spectrometry (UPLC-MS/MS), following a method adapted from Noga *et. al.*[1] This approach involved derivatization using the AccQ-Tag™ Ultra derivatization kit procured from Waters Corporation (Etten-Leur, the Netherlands). Plasma samples (5 µL) were prepared by spiking with an internal standard solution (30 µL) composed of a mix of $^{13}C^{15}N$-labeled amino acid analogs. Protein precipitation was achieved by the addition of 75 µL of MeOH, following which samples were centrifuged to isolate and dry the resulting supernatant in a SpeedVac. The residue was reconstituted in 80 µL of the borate buffer (pH 8.8) with 20 µL of AQC reagent for derivatization. After derivatization, samples were heated for 30 min at 55ºC at a speed of 900 rpm on an incubating microplate

shaker, following which they were transferred to autosampler vials and cooled to 4°C until injection into the UPLC-MS/MS system. An Agilent 1290 Infinity II System equipped with a Waters AccQ-Tag Ultra C18 column (2.1 × 100 mm, 1.7 µm) was used for the chromatographic separation of analytes from an injection volume of 1 µL. Targets were detected and measured in the positive electrospray ionization (ESI) mode using multiple reaction monitoring (MRM) with nominal mass resolution in an AB Sciex QTRAP 6500 triple-quadruple MS. Peak integration of the acquired MRM data was performed using the vendor software Sciex MultiQuant (v3.0.2).

S2.2 Signaling Lipids

Lipids involved in signaling pathways were measured in plasma using reversed-phase UHPLC-MS/MS, adapting the protocol published by Yang *et. al.*,[2] which employed two chromatographic methods to cover a wide range of compound classes; a low pH method for various oxylipins (such as prostaglandins and isoprostanes), endocannabinoids, and bile acids, and a high pH method for lysosphingolipids, lysoglycerophospholipids, and fatty acids ranging from C14 to C22 chain length species. Sample preparation involved spiking plasma samples (50 µL) with 5 µL of antioxidant solution (0.2 mg mL^{-1} BHT and 0.2 mg mL^{-1} EDTA), 5 µL of internal standard solution composed of deuterated target compounds, and 50 µL of 0.2 M citric acid and 0.4 M disodium hydrogen phosphate buffer (pH 4.5). Lipid extraction was performed using 400 µL of Butanol:MTBE (1:1 v/v) liquid-liquid extraction method. 350 µL of resulting organic phase was isolated, concentrated by drying in a SpeedVac, and reconstituted in a 50 µL of 70:30 (v/v) mixture of methanol and acetonitrile (MeOH:ACN) before transfer to autosampler vials for injection into the UPLC-MS/MS system. For chromatographic separation, a Shimadzu LC formed by three high pressure pumps (LC-30AD), communication module (CBM-20A), autosampler (SIL-30AC), and an oven (CTO-30A) from Shimadzu Benelux was utilized, employing a Waters BEH C18 column (2.1 × 50 mm, 1.7 µm) for the low pH analysis (10 µL injection volume) and a Kinetex EVO C18 column (2.1 × 50 mm, 1.7 µm) for the high pH analysis (5 µL injection volume). ESI-MS was conducted using a Sciex QTRAP 7500 triple-quadrupole MS for low pH analysis and a Sciex QTRAP 6500+ MS for high pH analysis, with polarity switching and dynamic MRM. Peak integration of the acquired MRM data was performed using the vendor software Sciex OS (v2.1.6.59781).

S3. Data Preprocessing

Quality control was conducted with an in-house software using pooled study samples, method blanks, and internal standards. Peak area ratios were computed using the closest eluting internal standard to correct for variations in sample preparation and instrument response. Metabolites with a relative standard deviation <30% in quality control

samples prepared by pooling study samples and background signal <40% in method blanks were included for downstream analysis, resulting in 254 of the 317 measured targets. Metabolites with missing data were examined using Fisher's exact test to assess whether missingness was associated with specific groups, and metabolites exceeding 20% missingness were excluded. Additionally, 66 biologically pertinent metabolite ratios and 16 sums (or means) were computed to provide insights into enzyme activities and shifts in metabolic pathways. A list of all included metabolic features and their abbreviations is provided in Table S1.

S4. Statistics

All descriptive statistics, statistical tests, and visualization were performed in RStudio (v4.3.1). Univariate differential analysis was performed on the comprehensive dataset using linear mixed-effect models (LMMs), via the lme4 package (version 1.1.34) with metabolite as the response, a dichotomous variable representing the comparison groups as the predictor, and patient identifier as random effects (1 | patient identifier) to account for within-individual variability. The models were corrected for clinical confounders identified by Fisher's exact test for categorical variables, Welch's t-test for continuous variables, and visual inspection by Principal Component Analysis (PCA). LMMs were weighted by the inverse number of samples per patient to down weigh oversampled patients. P-values for the LMMs were obtained using the lmerTest package via the Satterthwaite's degrees of freedom method. Two primary models were implemented to elucidate the similarities and differences between SINS and sepsis. First, the SINS and sepsis cases were combined into the "Inflamed" group and compared with controls to identify systemic inflammation-associated traits independent of etiology. Second, the control and SINS cases were combined into the non-sepsis group and compared with the "Sepsis" group to isolate sepsis-specific signatures amid systemic inflammation-associated traits. Additionally, an ANOVA across the three groups was performed, complemented by LMMs of pairwise comparisons: (i) control versus SINS (C_SINS model), (ii) control versus sepsis (C_S model), and (iii) SINS versus sepsis (SINS_S model) to validate the results of the two larger models and ensure an accurate interpretation of metabolic alterations by determining group-driven associations. Additional analyses investigated the combination of control and SINS vs sepsis, i.e., the Sepsis model to datasets stratified by sex, pathogen, and blood culture result. LMM outcomes were visualized using volcano plots and compared using forest plots and directed p-value plots, where the directed p-values were obtained by multiplying the negative logarithm (base 10) of the p-value with the direction of the estimate. ANOVA was performed on log2-transformed data prior adjusting for confounding factors, while mean fold change (FC) was calculated on untransformed data and considered when exceeding the RSD-defined technical variation from pooled study samples.

Univariate logistic regression analyses were conducted using generalized linear models with a logit link function on the STP dataset to investigate the potential of metabolites as early diagnostic biomarkers for sepsis. The binary response variable represented the comparison groups (control vs. SINS, control vs. sepsis, SINS vs. sepsis), with metabolite as predictor adjusted for the confounders stated earlier. These models yield insights into the association between metabolite levels and the likelihood or log odds of belonging to a specific group. Subsequently, multivariable diagnostic metabolomic models were developed to distinguish between SINS and sepsis at the timepoint of sepsis suspicion. A bootstrap-aggregated LASSO logistic regression, with 100 bootstrap iterations, was employed to mitigate overfitting and reduce the risk of data leakage given the limited sample size (n=57). In each iteration, samples were drawn with replacement to form the training set, while the remaining out-of-bag samples served as an internal test set. LASSO regression was performed within the training set via the glmnet package (version 4.1.8) with the *cv.glmnet* function, incorporating a 10-fold cross-validation to optimize the regularization parameter (λ). Model performance was evaluated on out-of-bag test samples, and metrics including area under the curve (AUC), sensitivity, and specificity were averaged across all iterations to estimate overall predictive performance. Robust features were identified by calculating the frequency of variable selection across all bootstraps. Metabolites selected in >45% of bootstrap models were retained to build a reduced, targeted logistic regression model. This final metabolomic model was evaluated using Leave-One-Out-Cross-Validation, thereby deriving the Area Under the Receiving Operating Characteristics Curve (AUROC) to assess the predictive performance parameters of the model. Additional models incorporating individual routine clinical inflammatory markers, combinations of the inflammatory markers, and combinations of the inflammatory markers and metabolites were assessed to compare the metabolomic model to conventional clinical diagnostic practices for sepsis detection. The McNemar's test was applied to the paired predictions from the reduced metabolite-based and inflammatory marker models to assess statistically significant differences in classification performance. Additionally, spearman correlations were calculated between diagnostic panel metabolites and inflammatory markers to provide complementary information. To further understand how these relationships shift under different inflammatory conditions, differential correlations were computed using the *compcorr* function from the DiffCorr package. A significance threshold of 0.05 was applied to p-values across all statistical analyses. To account for multiple testing, p-values were adjusted using Benjamini-Hochberg method implemented by the *p.adjust* function. These adjusted p-values, termed q-values, were subjected to a significance threshold of 0.1. These corrections accounted for the total number of metabolic features in univariate tests (n=296).

S5. Univariate Logistic Regression Model Results

Univariate analysis assessing the diagnostic utility of metabolites by determining the strength and direction of association between metabolites and odds of the condition, identified 61 metabolites with $p<0.05$ in the C_S model, of which 26 retained statistical significance after multiple testing correction ($q<0.1$). No statistically significant metabolites were observed in the C_SINS and SINS_S models, potentially due to reduced sample size within the STP dataset. However, 47 metabolites in the C_SINS model and 14 metabolites in the SINS_S model showed potential diagnostic utility ($p<0.05$; $q>0.1$). A comparison between the C_S and C_SINS models revealed that 31 of the 61 metabolites were exclusively associated with sepsis (Figure S5a). Several of these metabolites were also identified as differentially regulated in sepsis in the univariate analysis. Notably, phenylalanine/tyrosine (log OR: 4.46; 95% CI: 2.37-7.57), BCAA/AAA (log OR: 4.06; 95% CI: 1.69-7.03), LPE(16:0) (log OR: 3.25; 95% CI: 1.40-5.63), and LPI(16:0) (log OR: 4.17; 95% CI: 2.05-7.28), were associated with higher odds of sepsis, while 3-methoxy-tyrosine (log OR: -1.75; 95% CI: -3.22- -0.53), citrulline (log OR: -1.88; 95% CI: -3.51- -0.57), glutamine/glutamate (log OR: -1.20; 95% CI: -2.23- -0.31), 4-hydroxyproline/proline (log OR: -2.92; 95% CI: -5.18- -1.10), and sum of OG (log OR: -1.20; 95% CI: -2.30- -0.30) were associated with lower odds of sepsis, when compared to control. Furthermore, analysis of the SINS_S model revealed metabolites such as cortisol (log OR: 1.08; 95% CI: 0.15-2.35), putrescine (log OR: 1.60; 95% CI: 0.36-3.32), 11beta-PGF2alpha/AA (log OR: 0.91; 95% CI: 0.16-1.85), S1P(18 :2) (log OR: -2.41; 95% CI: -5.17- -0.28), and LPA(20.4) (log OR: -1.70; 95% CI: -3.56- -0.22) as potential biomarkers for distinguishing between SINS and sepsis (Figure S5b). Details of these logistic regression models are documented in Tables S12-S14.

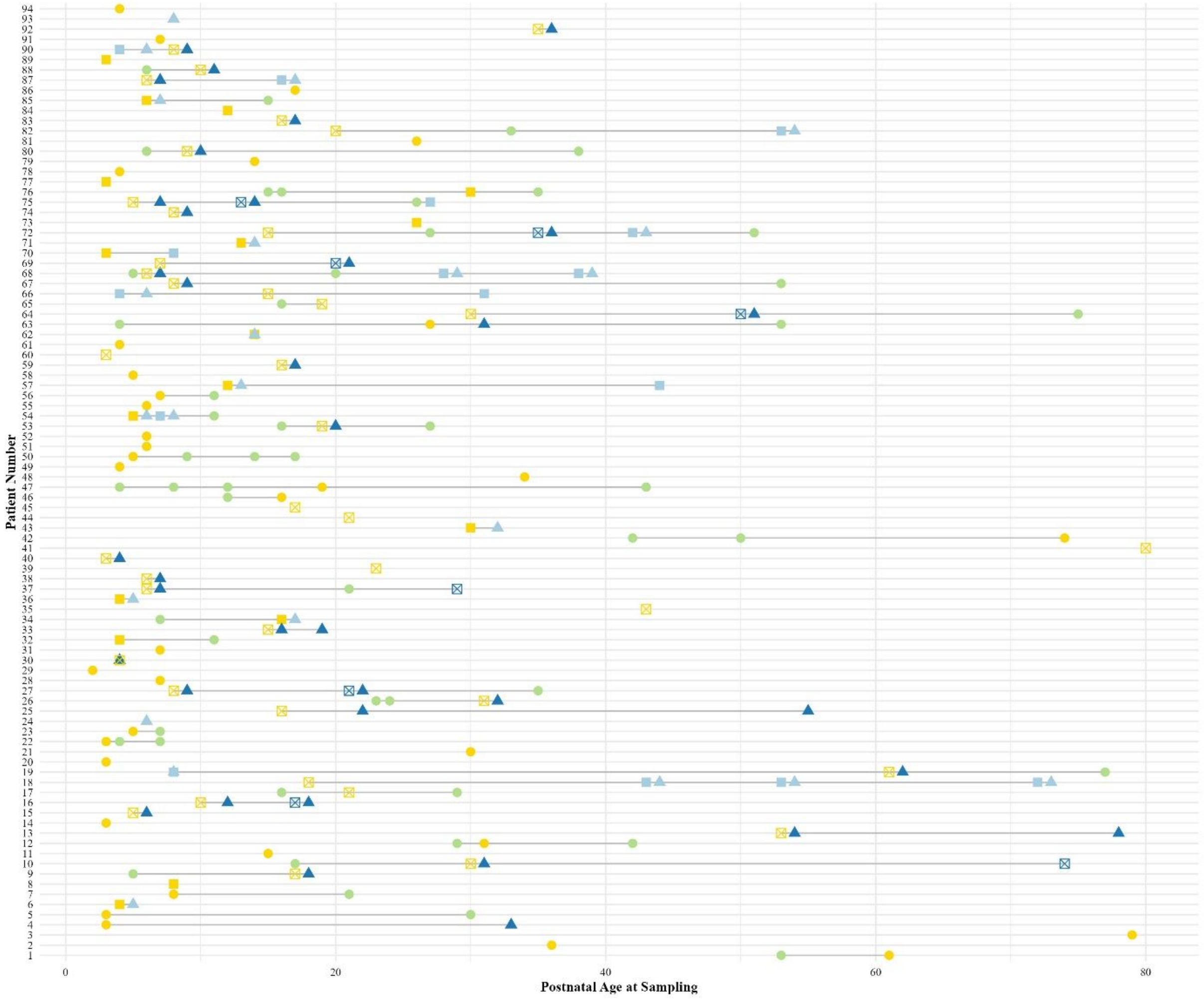

Figure S1. Temporal distribution of samples in the comprehensive and single time point datasets. Samples from each patient are plotted against the postnatal age at the time of sampling, with colors and shapes representing different groups: control (*green*; *filled circle*), systemic inflammation-no sepsis (*light blue*; *filled square*), sepsis (*blue*; *square with a cross*), and follow up (*filled triangle*). The single time point dataset was curated by selecting one sample per patient, prioritizing sepsis samples, followed by systemic inflammation-no sepsis, and including first episodes in multi-episode cases. All matched controls from patients without sepsis or systemic inflammation-no sepsis were also included. The selected single time point samples are highlighted in *gold*.

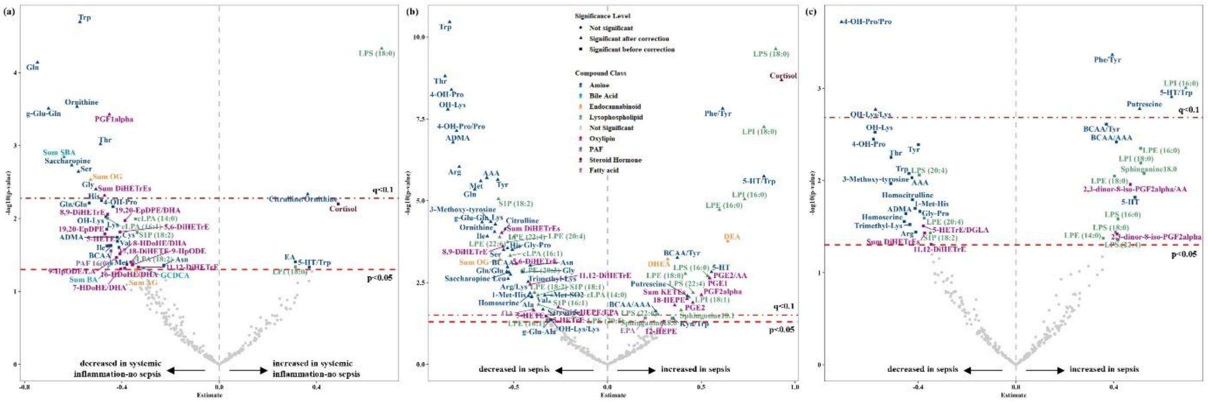

Figure S2. Differentially regulated metabolites in the C_SINS, C_S, and SINS_S models. The figure presents volcano plots for the C_SINS (a), C_S (b), and SINS_S (c) models, showcasing the differential expression of metabolites by depicting the relationship between the estimate (x-axis) and statistical significance (-log10 p-value, y-axis). Metabolites are color-coded by compound classes, including amines, bile acids, endocannabinoids, fatty acids, lysophospholipids, oxylipins, steroid hormones, and PAF. Data points are shaped based on significance levels: significant after FDR correction, significant before FDR correction, and not significant. The vertical dashed line indicates an estimate of zero effect, and horizontal lines denote significance thresholds. FDR = False Discovery Rate; PAF = Platelet Activating Factor; C_SINS = control versus systemic inflammation-no sepsis; C_S = control versus sepsis; SINS_S = systemic inflammation-no sepsis versus sepsis.

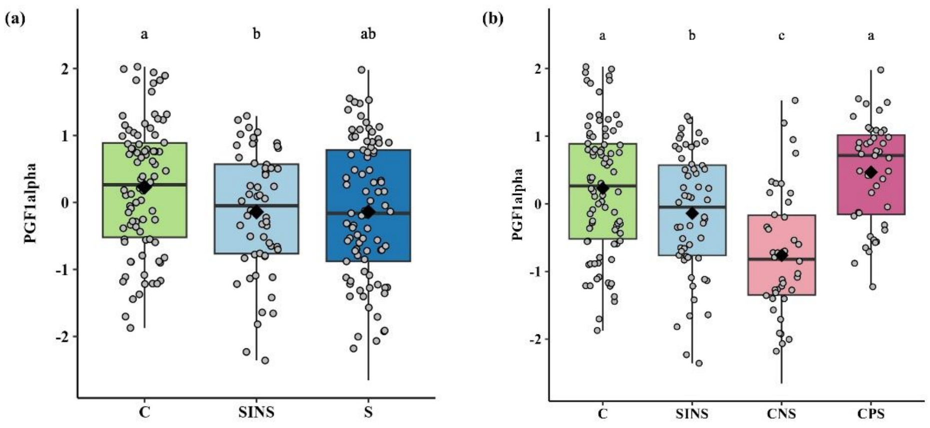

Figure S3. Differential regulation of PGF1alpha across SINS and sepsis subgroups. The figure presents a series of boxplots illustrating the distribution of PGF1alpha levels prior to confounder correction. (a) Boxplots illustrating the significant downregulation of PGF1alpha exclusively in the SINS group compared to controls, showing its distinct regulation in systemic inflammation without sepsis. (b) Boxplots depicting the stratification of the sepsis group by blood culture outcome, revealing contrasting patterns of PGF1alpha regulation, with significant downregulation in culture-negative sepsis and an upward trend in culture-positive sepsis. In each boxplot, the central line represents the mean, with box edges denoting the interquartile range. Individual data points represent samples, and the black diamond represents the mean. The letters denote significance between groups from the linear-mixed effect models: shared letters indicate no significant difference, while unique letters indicate significant difference. C = Control (*green*); SINS = Systemic Inflammation-No Sepsis (*light blue*); S = Sepsis (*blue*); CNS = Culture-Negative Sepsis (*light pink*); CPS = Culture-Positive Sepsis (*dark pink*).

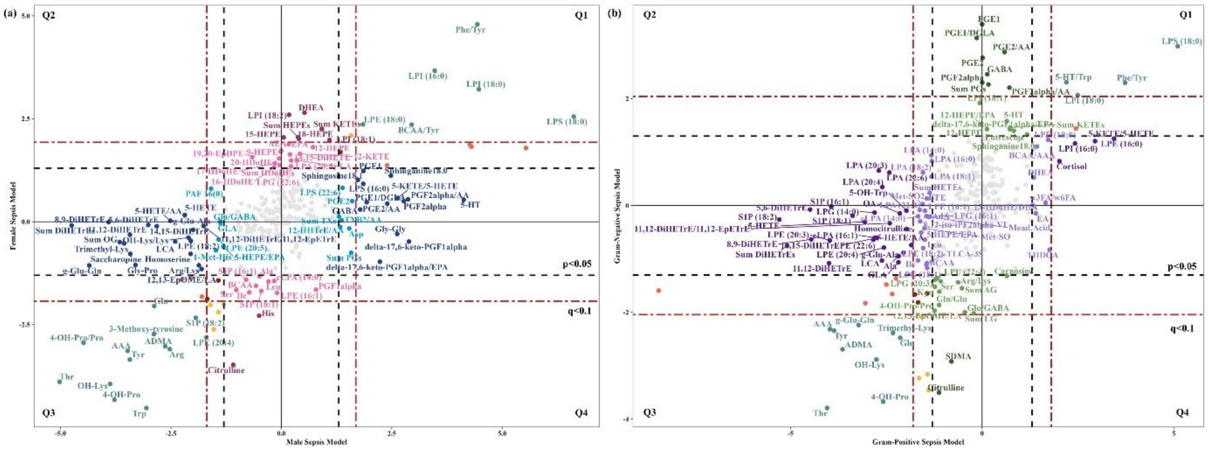

Figure S4. Comparative analysis of differentially regulated metabolites in sex- and pathogen-stratified linear mixed-effect sepsis models. The figure presents the directed p-value plots of the sex and pathogen-stratified analysis. The left panel (a) showcases the sex-stratified analysis, plotting the directed p-values of the male sepsis model in the x-axis and the directed p-values of the female sepsis model on the y-axis, color-coded based on significance level, before and after FDR correction (*dark pink*: significant after correction exclusively in female; *light pink*: significant before correction exclusively in female; *dark blue*: significant after correction exclusively in male; *light blue*: significant before correction exclusively in male; *slate gray*: significant after correction both in male and female). Metabolites in Q1 are increased in both males and females, those in Q2 are decreased in males and increased in females, those in Q3 are decreased in both males and females, and those in Q4 are increased in males and decreased in females. The right panel (b) displays the pathogen-stratified analysis, plotting the directed p-values of the gram-positive sepsis model in the x-axis and the directed p-values of the gram-negative sepsis model on the y-axis, color-coded based on significance level, before and after FDR correction (*dark purple*: significant after correction exclusively in gram-positive; *light purple*: significant before correction exclusively in gram-positive; *dark green*: significant after correction exclusively in gram-negative; *light green*: significant before correction exclusively in gram-negative; *slate gray*: significant after correction both in gram-negative and gram-positive). Metabolites in Q1 are increased in both gram-positive and gram-negative sepsis, those in Q2 are decreased in gram-positive and increased in gram-negative sepsis, those in Q3 are decreased in both gram-positive and gram-negative sepsis, and those in Q4 are increased in gram-positive and decreased in gram-negative sepsis. FDR = False Discovery Rate; Q = Quadrant.

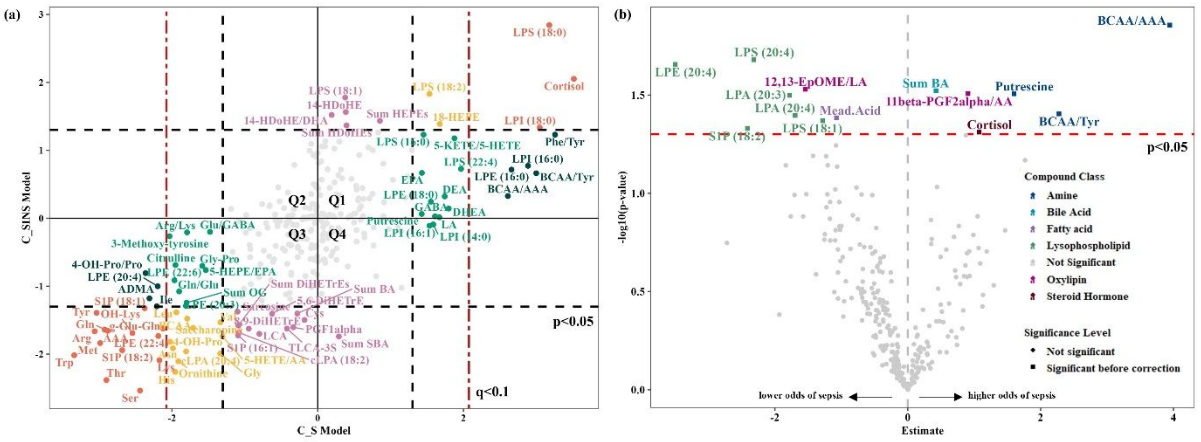

Figure S5. Diagnostic potential of metabolites derived from univariate logistic regression analysis. The left panel (a) presents a directed p-value plot comparing the logistic regression estimates between the control versus sepsis (C_S) and the control versus systemic inflammation-no sepsis (C_SINS) models from the single time point dataset. A unit increase in the metabolites present in Q1 increases the odds of both sepsis and systemic inflammation-no sepsis, those in Q2 decreases the odds of sepsis but increases the odds of systemic inflammation-no sepsis, those in Q3 decreases the odds of both sepsis and systemic inflammation-no sepsis, and those in Q4 increases the odds of sepsis but decreases the odds of systemic inflammation-no sepsis. Metabolites are color-coded based on significance, before and after FDR correction (*dark green*: significant after correction exclusively in C_S model; *light green*: significance before correction exclusively in C_S model; *pink*: significant before correction exclusively in C_SINS model; *yellow*: significant before correction in both C_S and C_SINS models; *orange*: significant after correction in C_S model and before correction in C_SINS model). The right panel (b) features a volcano plot of the systemic inflammation-no sepsis versus sepsis (SINS_S) model highlighting the direction and significance of metabolic associations with sepsis compared to systemic inflammation-no sepsis. These visualizations provide insights into the differential metabolic profiles and their potential diagnostic value in distinguishing between the two inflammatory conditions. C_S = control versus sepsis; C_SINS = control versus systemic inflammation-no sepsis; SINS_S = systemic inflammation-no sepsis versus sepsis; FDR = False Discovery Rate; Q = Quadrant.

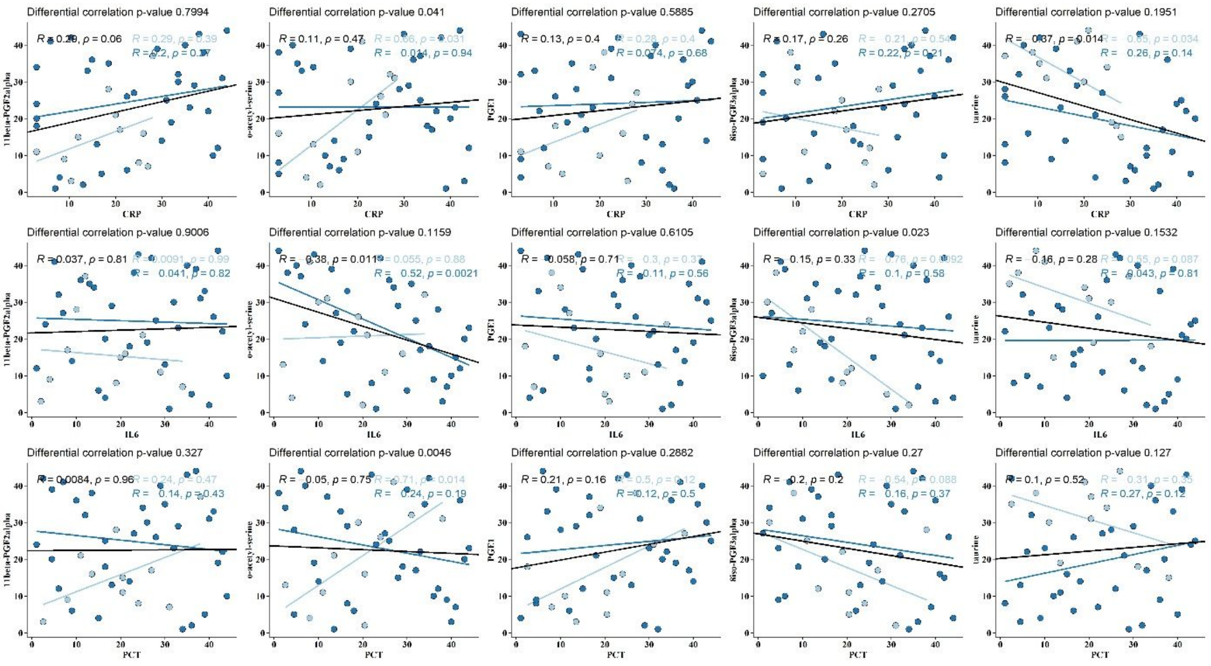

Figure S6. Spearman correlation analyses of the metabolites in the diagnostic panel with the routine inflammatory markers. The figure presents the scatter plots of the ranks of five metabolites in the diagnostic panel with the ranks of the three routine inflammatory markers. Each plot illustrates the correlation coefficient rho and p-value for all datapoints combined, as well as stratified by group (systemic inflammation-no sepsis (*light blue*) and sepsis (*blue*)). The differential correlation p-value is also displayed in each plot to highlight significant differences between the two groups. BCAA: Branched Chain Amino Acids; AAA: Aromatic Amino Acids.

REFERENCES

1. Noga MJ, Dane A, Shi S, Attali A, van Aken H, Suidgeest E, et al. Metabolomics of cerebrospinal fluid reveals changes in the central nervous system metabolism in a rat model of multiple sclerosis. Metabolomics. 2012;8(2):253–63.

2. Yang W, Schoeman JC, Di X, Lamont L, Harms AC, Hankemeier T. A comprehensive UHPLC-MS/MS method for metabolomics profiling of signaling lipids: Markers of oxidative stress, immunity and inflammation. Anal Chim Acta. 2024 Apr 8;1297:342348.
